# Hybrid sequencing reveals insight into heat sensing and signaling of bread wheat

**DOI:** 10.1111/tpj.14299

**Published:** 2019-04-23

**Authors:** Xiaoming Wang, Siyuan Chen, Xue Shi, Danni Liu, Peng Zhao, Yunze Lu, Yanbing Cheng, Zhenshan Liu, Xiaojun Nie, Weining Song, Qixin Sun, Shengbao Xu, Chuang Ma

**Affiliations:** ^1^ State Key Laboratory of Crop Stress Biology for Arid Areas College of Agronomy Northwest A&F University Yangling 712100 Shaanxi China; ^2^ State Key Laboratory of Crop Stress Biology for Arid Areas College of Life Sciences Northwest A&F University Yangling 712100 Shaanxi China; ^3^ Center of Bioinformatics College of Life Sciences Northwest A&F University Yangling 712100 Shaanxi China; ^4^ Frasergen Wuhan East Lake High‐tech Zone Wuhan 430075 China; ^5^ Department of Plant Genetics & Breeding China Agricultural University Yuanmingyuan Xi Road No. 2, Haidian District Beijing 100193 China

**Keywords:** heat sensing and signaling, early heat stress, hybrid sequencing, spatio‐temporal transcriptome, transcriptional regulation, alternative splicing regulation, wheat (*Triticum aestivum* L.)

## Abstract

Wheat (*Triticum aestivum* L.), a globally important crop, is challenged by increasing temperatures (heat stress, HS). However its polyploid nature, the incompleteness of its genome sequences and annotation, the lack of comprehensive HS‐responsive transcriptomes and the unexplored heat sensing and signaling of wheat hinder our full understanding of its adaptations to HS. The recently released genome sequences of wheat, as well as emerging single‐molecular sequencing technologies, provide an opportunity to thoroughly investigate the molecular mechanisms of the wheat response to HS. We generated a high‐resolution spatio‐temporal transcriptome map of wheat flag leaves and filling grain under HS at 0 min, 5 min, 10 min, 30 min, 1 h and 4 h by combining full‐length single‐molecular sequencing and Illumina short reads sequencing. This hybrid sequencing newly discovered 4947 loci and 70 285 transcripts, generating the comprehensive and dynamic list of HS‐responsive full‐length transcripts and complementing the recently released wheat reference genome. Large‐scale analysis revealed a global landscape of heat adaptations, uncovering unexpected rapid heat sensing and signaling, significant changes of more than half of HS‐responsive genes within 30 min, heat shock factor‐dependent and ‐independent heat signaling, and metabolic alterations in early HS‐responses. Integrated analysis also demonstrated the differential responses and partitioned functions between organs and subgenomes, and suggested a differential pattern of transcriptional and alternative splicing regulation in the HS response. This study provided comprehensive data for dissecting molecular mechanisms of early HS responses in wheat and highlighted the genomic plasticity and evolutionary divergence of polyploidy wheat.

## Significance Statement

Hybrid sequencing illustrates the spatio‐temporal landscape of heat adaptations in wheat filling grain and flag leaves at isoform resolution, providing the most comprehensive heat‐responsive transcripts to date. These data demonstrate that heat sensing and signaling are more rapid than previously assumed, they uncover the earliest heat‐responsive events, and highlight the evolutionary divergence and advantages of the polyploid nature of wheat in environmental adaptations.

## Introduction

Wheat (*Triticum aestivum* L.), the most widely cultivated crop, contributes about one‐fifth of the total calories consumed by humans (Appels *et al*., 2018). The growth, yield and quality of wheat are adversely affected by global climate changes, particularly increasing temperatures (heat stress, HS) (Tingley and Huybers, 2013; Lobell and Tebaldi, 2014; Tack *et al*., 2015; Lesk *et al*., 2016). Each degree Celsius increase in global mean temperature would reduce wheat yield by 6.0% (Zhao *et al*., 2017), and this reduction is most severe during the grain‐filling stage (Wollenweber *et al*., 2003; Farooq *et al*., 2011; Tack *et al*., 2015; Lesk *et al*., 2016; Wang *et al*., 2018a). A comprehensive understanding of the heat responses and adaptations, especially at the grain‐filling stage, is therefore urgently required to enable the development of practices to improve wheat thermotolerance.

Previous studies have shown that the heat signal is probably transduced by several pathways to converge on heat shock factors (HSFs), followed by activation of a number of heat shock proteins (HSPs) and other heat‐responsive genes that drive the heat adaptation process in plants (Kotak *et al*., 2007; Saidi *et al*., 2011; Bokszczanin *et al*., 2013). This heat adaptation confers thermotolerance on plant growth and development, and majorly contributes to maintaining the grain‐filling rate and the final yield of crops under HS (Wang *et al*., 2018a). However, how plants sense HS and which pathways are involved in heat sensing and signaling to activate heat adaptation are largely unknown, hindering our full understanding of plant thermotolerance. Previously, short‐term HS‐response events have been explored by analyzing transcriptomes after 15 and 30 min heat treatments in rice and barley, respectively (Mangelsen *et al*., 2011; Wilkins *et al*., 2016). Significantly, our recent study showed that some markers of the HS response (e.g., small heat shock proteins [sHSPs]) were highly upregulated after 10 min HS treatment in wheat (Wang *et al*., 2017b), implying that more intensive studies involving the analysis of earlier HS treatment samples should be considered to uncover heat sensing and early signaling events. We also identified extensive alternative splicing (AS) events in HS‐response transcriptomes in wheat seedlings (Liu *et al*., 2018), revealing the importance of post‐transcriptional regulation in HS adaptation in wheat. However, existing transcriptome analyses have been based on incomplete genome sequences, incomprehensive gene annotation, a lack of grain‐filling stage samples and fragmented genes assembled from second‐generation sequencing data (usually 100–150 base pairs [bp]), making it impossible to capture the global landscape of HS responses and adaptations in wheat.

The allohexaploid nature, large genome (~17 gigabases) and existence of three highly similar subgenomes (A, B, and D) of bread wheat have hindered the availability of high‐quality reference genome sequences. Most recently, the International Wheat Genome Sequencing Consortium (IWGSC) presented an ordered and annotated assembly of the 21 chromosomes of the allohexaploid wheat cultivar Chinese Spring (IWGSC RefSeq v1.0) (Appels *et al*., 2018), providing the most comprehensive wheat genome sequences published to date with which to investigate the molecular mechanisms of heat and other stresses. However, wheat genome annotation is still in the nascent phase and requires further improvements in terms of transcript length and AS isoforms (as shown in this study). For transcript recovery and isoform detection, second‐generation sequencing technology (e.g., Illumina sequencing), which features high‐throughput capability and provides high‐quality reads, is an option. However, its limitations, in particular the short read length, mean that computational assembly is required, therefore potentially introducing errors (Rhoads and Au, 2015; Goodwin *et al*., 2016). While in allohexaploid wheat, highly similar subgenomes and the higher proportion of homologous genes pose a larger challenge. Single‐molecule real‐time sequencing technology, also known as third‐generation sequencing (e.g., PacBio sequencing), can directly generate full‐length transcripts and is, therefore, well suited for transcript recovery and isoform detection in species with well sequenced and/or incomplete genome sequences (Abdel‐Ghany *et al*., 2016; Wang *et al*., 2016, 2017a). However, the higher error rate and low throughput have hindered the widespread application of this methodology (Rhoads and Au, 2015).

**Figure 1:**
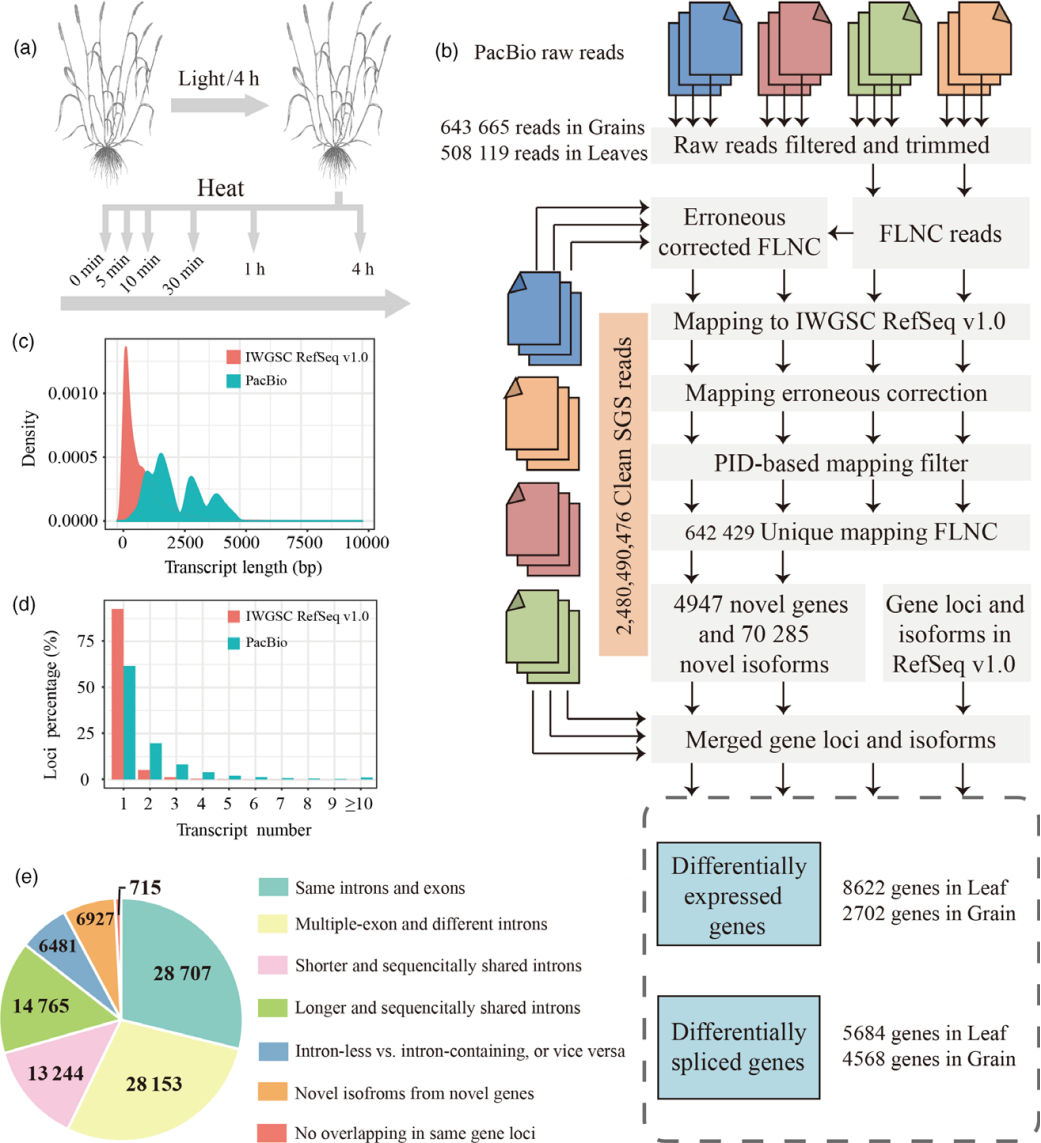
Experimental workflow. (a) Wheat plants (*T. aestivum* cv. Chinese Spring) at 15 days after anthesis were subjected to heat stress (HS) (37°C). The flag leaves and filling grain were sampled at 0 min, 5 min, 10 min, 30 min, 1 h and 4 h under HS. In total, 36 samples (six time points for each of the two organs, three biological replicates per time point) were sequenced using second‐generation sequencing, and two mixed samples (the RNAs of 18 samples from each organ mixed in equal volume) were sequenced using third‐generation sequencing. (b) Bioinformatics pipeline for analyzing the hybrid sequencing data. Sequencing errors in full‐length and non‐chimeric (FLNC) reads were corrected with short reads. FLNC reads before and after error correction were mapped in parallel to IWGSC RefSeq v1.0, and the read with the best genomic match (see Experimental procedures) was retained in the downstream analysis. (c) Comparison of the transcript length between the IWGSC RefSeq v1.0 annotation and the PacBio data. (d) Comparison of the isoform number between the IWGSC RefSeq v1.0 annotation and the PacBio data. (e) Structure comparison of the IWGSC RefSeq v1.0 and PacBio transcripts.

To address these issues, we proposed a hybrid sequencing strategy to generate a high‐resolution spatio‐temporal transcriptome map of wheat for exploring heat sensing and signaling in relatively early stress response events (Figure 1a,b). The hybrid sequencing strategy takes advantages of both second‐generation sequencing and third‐generation sequencing, without the requirement for computational assembly of Illumina short reads and overcoming the limitations of long reads with higher error rates and low throughput. We introduced two percentage‐of‐identity (PID)‐related measures (Figure S1), local PID and global PID, to map long reads onto the genome of allohexaploid wheat at subgenome resolution. Based on this valuable resource, we systematically investigated heat sensing, signaling transduction and heat adaptation processes at multidimensional levels, providing a comprehensive understanding of the rapid and dynamic HS responses in wheat grain and leaves and unveiling the differential responses between organs and subgenomes. Our results greatly facilitate dissection of the complex network of mechanisms underlying heat adaptation in plants and provide guidance for other abiotic stress studies and for the analysis of hybrid sequencing data.

## Results

### Spatio‐temporal transcriptome profiling of wheat flag leaves and filling grain during the early HS response

To comprehensively investigate the wheat transcriptomes present during the early HS response, we sequenced 36 mRNA samples from the flag leaves and grain of *T. aestivum* cv. Chinese Spring that had been subjected to HS for 0 min, 5 min, 10 min, 30 min, 1 h and 4 h using the HiSeq X Ten platform (second‐generation sequencing). In total, 2 480 490 476 paired‐end short reads were yielded for all samples (Table S1). RNAs from samples of the same organ were then equally mixed for single‐molecule real‐time sequencing using the PacBio RS II platform (third‐generation sequencing), yielding 643 665 and 508 119 circular consensus sequence reads for grain and leaves, respectively (Table S1). From these circular consensus sequence reads, full‐length and non‐chimeric (FLNC) reads were identified based on the inclusion of 5′ primer, 3′ primer, and 3′ poly(A) tails, followed by error correction using Illumina short reads, FLNC reads mapping and a filtration procedure (see Experimental procedures). This left 705 335 high‐quality FLNC reads, 91.08% of which were uniquely mapped to the IWGSC RefSeq v1.0 (Table S2).

The uniquely mapped FLNC reads were collapsed into 98 992 non‐redundant transcripts, the mean length of which (2410 bp) was higher than that of transcripts annotated in IWGSC RefSeq v1.0 (993 bp) (Figure 1c and Table S3). These 98 992 transcripts were derived from 51 160 gene loci, covering 46.18% of the high‐confidence genes annotated in IWGSC RefSeq v1.0. Of the 51 160 identified gene loci, 45.18% were multiple‐exon genes encoding two or more isoforms (an average of 2.1 isoforms) compared with 21.87% of multiple‐exon genes with two or more isoforms in high‐confidence gene sets (Figure 1d). These results highlight that our hybrid sequencing strategy significantly improved wheat genome annotation in terms of the length and the quantity of transcripts.

### Characterization and verification of newly discovered loci and isoforms

**Figure 2:**
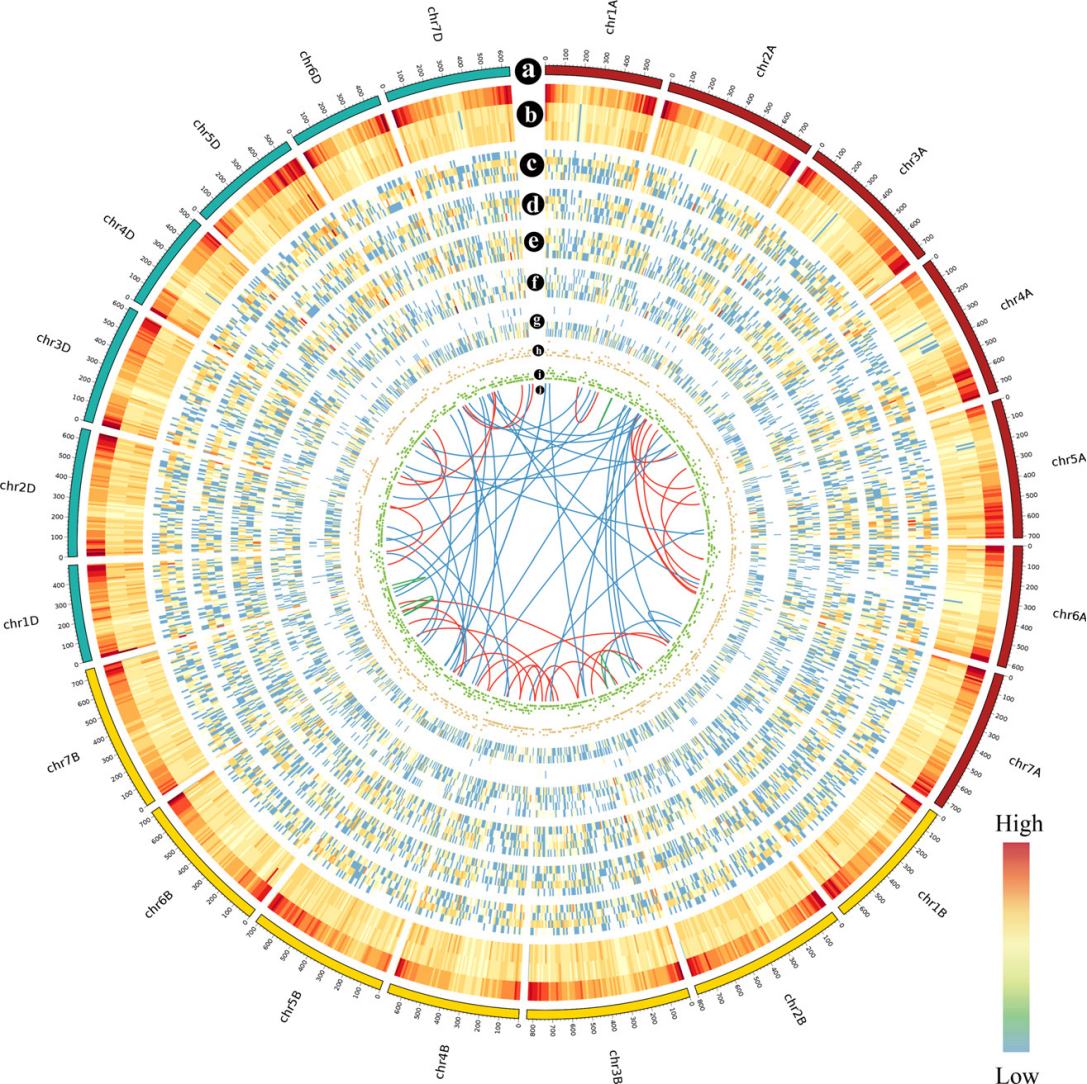
CIRCOS visualization of different data at the genome‐wide level. The density was calculated in a 10‐Mb sliding window. (a) Karyotype of the wheat genome. (b) Comparison of transcript density between the IWGSC RefSeq v1.0 annotation and the PacBio data. From the upper to lower tracks: transcripts in IWGSC RefSeq v1.0, transcripts in grain and transcripts in leaves. (c–g) Distribution of heat stress (HS)‐responsive genes following HS treatment for 4 h, 1 h, 30 min, 10 min and 5 min. From the upper to lower tracks in each part: the HS‐responsive genes in grain with transcriptional regulation, the HS‐responsive genes in leaves with transcriptional regulation, the HS‐responsive genes in grain with alternative splicing (AS) regulation and the HS‐responsive genes in leaves with AS regulation. (h, i) Distribution of lncRNAs in grain (h) and leaves (i). (j) Linkage of fusion transcripts: intra‐chromosome (green), inter‐chromosome in the same subgenome (red) and inter‐chromosome in different subgenomes (blue).

By comparison with the IWGSC RefSeq v1.0 annotation, PacBio transcripts could be classified into seven groups (Figures 1e and S2). In total, 4947 (9.67%) chromosomal loci were newly identified that did not overlap with any annotated genes in the IWGSC RefSeq v1.0, encoding 6927 isoforms (2774 were single‐exon and 4153 were multiple‐exon), and 63 358 isoforms (representing novel isoforms) from the 29 040 gene loci already annotated in the IWGSC RefSeq v1.0 (Figure 2 and Table S4). For the newly identified isoforms, 94.64% of their splice junctions were validated by aligning Illumina short reads to the wheat reference genome. Some of the newly discovered genes and isoforms were further validated using reverse transcription‐polymerase chain reaction (RT‐PCR) with the mixed RNAs extracted from the grain and leaf samples of Chinese Spring (Figure S3). These results indicated that the hybrid sequencing strategy generated a set of high‐quality wheat transcripts for the comprehensive analysis of HS responses in wheat.

Furthermore, we searched the newly discovered isoforms against the public databases and identified a total of 60.14% novel isoforms from unannotated loci and 96.98% novel isoforms from annotated genes with sequence similarity to previously annotated proteins (Table S5). The function and domain annotation showed that no certain functions or gene families were underrepresented or missed in the IWGSC RefSeq v1.0 (Figure S4 and Tables S6–S8). For the remaining newly discovered isoforms with no sequence similarity to previously annotated proteins, 2678 (38.66%) isoforms from unannotated loci and 1584 (2.81%) novel isoforms from annotated genes were characterized as long non‐coding RNAs (lncRNAs) (Table S9), highlighting the advantage of PacBio sequencing in identifying lncRNAs (Abdel‐Ghany *et al*., 2016; Wang *et al*., 2016) and providing a comprehensive lncRNA database in wheat.

### Identification of differentially expressed genes and differentially spliced genes during heat response in wheat flag leaves and filling grain

**Figure 3:**
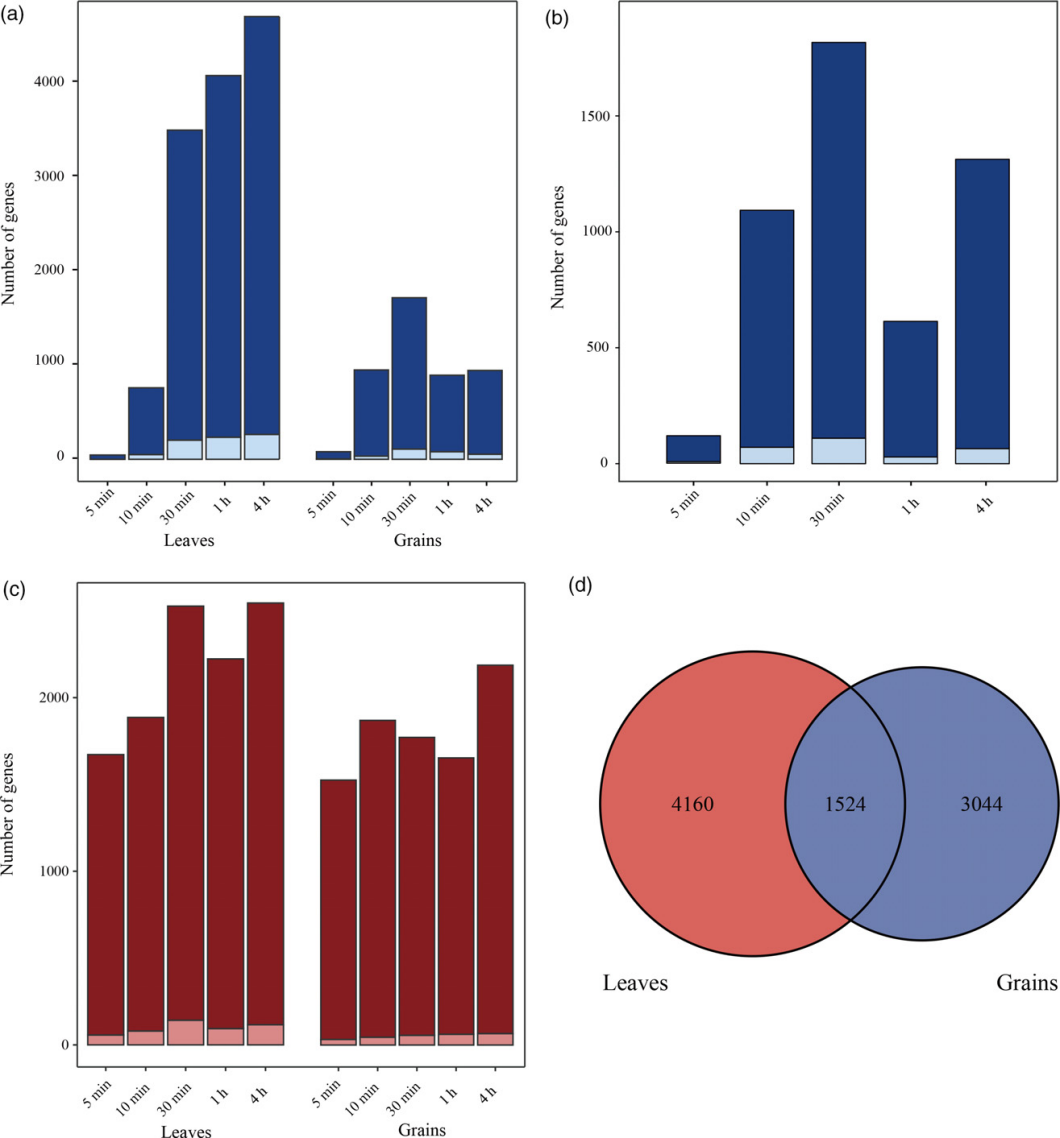
Identification of differentially expressed genes (DEGs) and differentially spliced genes (DSGs) at each time point in leaves and grain. Number of DEGs (a) and DSGs (c). The *x*‐axis represents the heat stress (HS) treatment time points and the *y*‐axis represents the HS‐responsive gene number. Light blue and light red represent the number of DEGs and DSGs that were newly discovered loci from the PacBio data, respectively. (b) Number of different HS‐response genes between leaves and grain. Light blue represents newly discovered loci from the PacBio data. (d) Venn diagram of DSGs in leaves and grain.

For each sample, the expression of annotated and newly discovered transcripts in terms of fragments per kilobase of exon per million fragments mapped (FPKM) was estimated from the corresponding alignments between the Illumina short reads and the IWGSC RefSeq v1.0. By comparison with the 0 min HS treatment sample, 8622 and 2702 genes, including the 468 and 144 newly discovered genes, were identified as differentially expressed genes (DEGs, fold change ≥2.0, FDR‐adjusted *P*‐value <0.05, FPKM ≥ 1) in at least one time‐point sample, accounting for 14.38% and 5.01% of the expressed genes (FPKM ≥ 1 for at least one time point) in leaves and grain, respectively (Figures 3a and S5 and Table S10), suggesting a distinct response pattern between leaves and grain. Results of principal component analysis and correlation analysis of the whole response transcriptomes also strongly supported this distinct HS‐response pattern (Figure S6). With the multiple factor analysis implemented in edgeR, we directly characterized 3153 genes as HS‐response genes that differed between leaves and grain (Figure 3b) and that were mainly over‐represented in the Gene Ontology (GO) terms ‘protein folding’, ‘response to stimulus’ and ‘protein binding’ and the Kyoto Encyclopedia of Genes and Genomes (KEGG) pathways ‘protein processing in endoplasmic reticulum’, ‘mitogen‐activated protein kinase (MAPK) signaling pathway’ and ‘spliceosome’ (Table S11), implying that leaves and grain employed distinct gene sets or varied in intensity in these well known processes in HS responses.

Taking into account the qualitative and quantitative data on the transcripts identified by our hybrid sequencing strategy, we analyzed AS alterations under HS. By comparing highly expressed transcripts and investigating changes in transcript expression, we identified, in total, 5684 (9.48% of the expressed genes) and 4568 (8.48% of the expressed genes) differentially spliced genes (DSGs), including 281 and 138 newly discovered genes, in leaves and grain, respectively (Figures 3c and S7 and Table S12). Like the different responses observed between leaves and grain in terms of transcriptional regulation, only 26.81% of DSGs in leaves and 33.36% of DSGs in grain overlapped (Figures 3d and S8). However, by contrast with the dramatically expanded number of DEGs over the duration of the HS, the number of DSGs remained relatively stable (Figure 3c), suggesting a different mode of AS regulation compared with transcriptional regulation.

In total, we identified 98 107 AS events distributed among 11 399 genes, including four main types: exon skipping (ES), intron retention (IR), alternative donor sites (AD), and alternative acceptor sites (AA), plus more complex types (Figure S9). IR is the primary mode of AS in plants and was markedly repressed in the HS response in moss (Chang *et al*., 2014; Shen *et al*., 2014). In our data, the IR ratio decreased from 49.19% in all PacBio isoforms to 44.83% in the isoforms generated by DSGs (Table S13), suggesting that IR was also repressed in the HS response in wheat and this phenomenon was common to moss and higher plants. Furthermore, the IR ratio further decreased from 44.83% to 34.17% in isoforms (FPKM ≥ 1.0) generated by DSGs, indicating that IR was further repressed in highly expressed isoforms of DSGs. Analysis of the expression levels of the four AS mode isoforms (FPKM ≥ 1.0) confirmed the lower expression levels of IR‐related isoforms (Figure S10). The reports that retained introns act widely to downregulate transcripts (Wong *et al*., 2013; Braunschweig *et al*., 2014) may account for this expression repression of IR‐related isoforms.

### Unexpectedly rapid heat‐induced DEGs and DSGs

**Figure 4:**
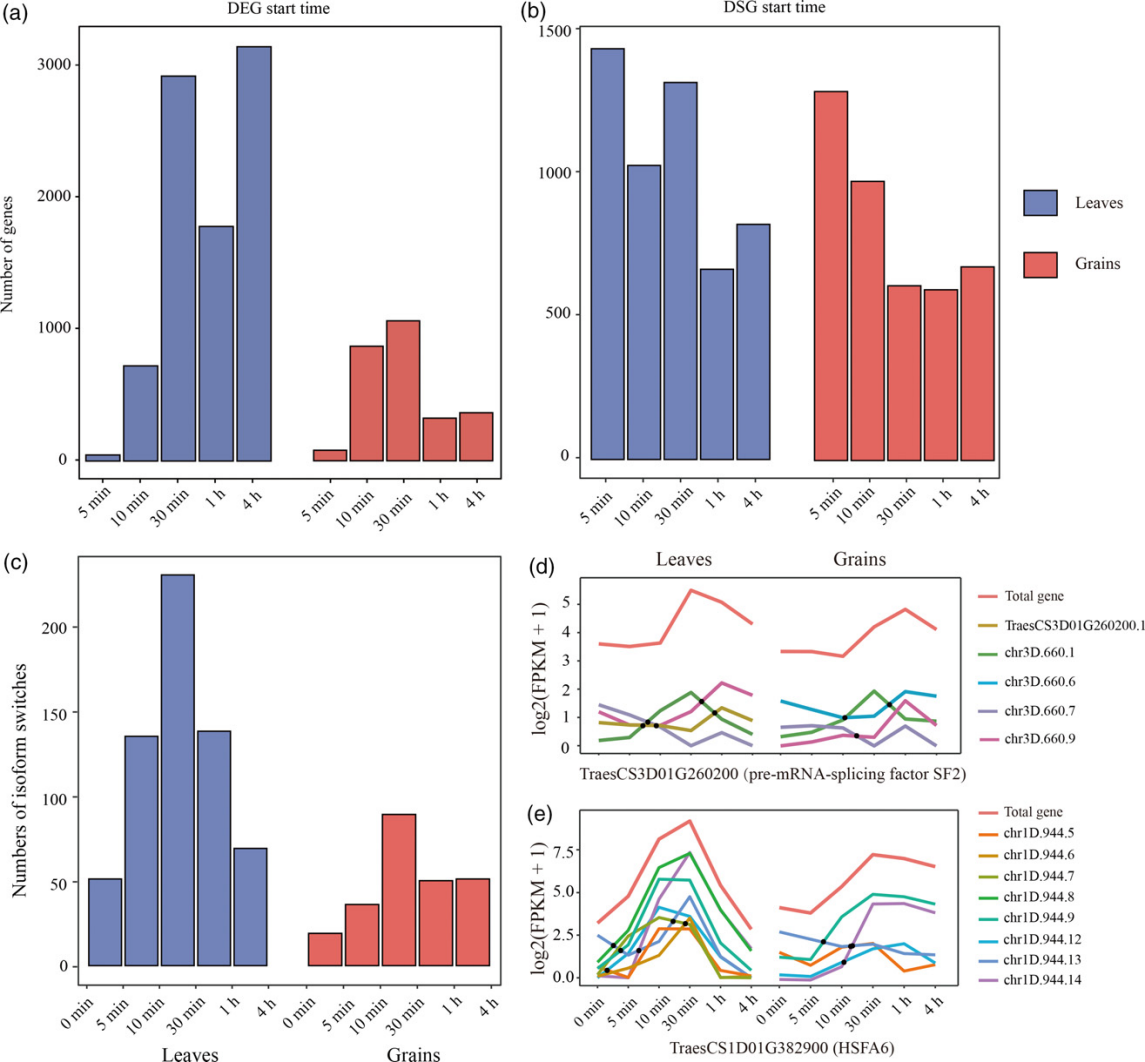
Rapid changes of differentially expressed genes (DEGs) and differentially spliced genes (DSGs) in response to heat stress (HS). (a, b) Histograms plots of the time points at which the DEGs (a) and DSGs (b) first showed a significant difference in leaves and grain. Each gene is represented only once in each histogram. (c) Frequency over time of isoform switches (where the relative abundance of different isoforms is reversed in response to HS) in the time‐course transcriptomes. (d, e) Expression profiles of pre‐mRNA‐splicing factor SF2 (d) and HSFA6 (e). The isoform switch events were marked with black circles. For clarity, only the transcripts that were involved in isoform switches were plotted. The isoforms whose names start with ‘chr’ were novel isoforms identified from the PacBio data.

The high‐resolution spatio‐temporal transcriptomes allowed us to determine the precise time point at which each DEG and DSG first showed a significant change (start time point), along with the magnitude and duration of that change. By plotting the distribution of start time points, we found that 42.83% and 74.50% of DEGs, and 71.78% and 69.31% of DSGs in leaves and grain were detected within the first 30 min of heat treatment, respectively (Figure 4a,b). Furthermore, the speed of the heat response was highlighted by 46 and 80 DEGs, and 1672 and 1526 DSGs in the leaves and grain after only 5 min of heat treatment (Figure 3a,c), indicating that heat sensing and signal transduction in plants is much quicker than previously thought (Mangelsen *et al*., 2011; Wilkins *et al*., 2016).

A further indicator of the speed of AS regulation was demonstrated by identifying those DSGs that underwent isoform switches (IS), where the relative abundance of different isoforms was reversed during the HS response, using the Time‐Series Isoform Switch (TSIS) program (Guo *et al*., 2017). In total, 623 and 245 ISs were identified in 274 and 162 DSGs in leaves and grain, respectively (Figure 4c–e). About 67.74% and 59.18% of the ISs occurred between 0 and 30 min following heat treatment. Furthermore, 8.35% and 7.76% of the ISs occurred between 0 and 5 min, in leaves and grain, respectively, further supporting the evidence that AS regulation occurs rapidly in response to HS.

As expected, we showed the rapid heat‐induction of HSFs and HSPs, which are well known marker genes of the HS response (Figures S11 and S12 and Tables S14 and S15). In total, three HSFs and 17 sHSPs, were differentially expressed in leaves at 5 min after the onset of heat treatment, whereas no HSFs and HSPs were detected as DEGs in grain at this time point. Up to the 10 min time point, four HSFs and 15 HSPs (14 belonging to sHSPs) were detected as DEGs in grain, suggesting that heat sensing and signaling transduction were slower in grain than in leaves. For AS regulation, among the DSGs at the 5 min time point, four HSFs (A6 subfamily) and eight HSPs (four belonging to sHSPs) were detected in leaves. In grain, similar to transcriptional regulation, no HSFs were observed in DSGs at the 5 min time point. However, five HSPs were identified as DSGs at 5 min in grain, indicating that the AS regulation of some HSPs that are known as typical targets of HSFs (Scharf *et al*., 2012; Wang *et al*., 2018b), may be HSF independent in the early HS response.

### Differences in timing of diverse transcription factors (TFs) in heat signaling

Transcription factors that perceive stress signals and activate the expression of stress‐responsive genes play master roles in gene regulatory networks under environmental stress (Hennig, 2012). The IWGSC RefSeq v1.0 contains 8908 annotated TFs belonging to 59 families, encoding 10 356 TF isoforms. Among our data, we identified 27 novel members of 13 families and 1640 novel isoforms of 52 families, increasing the number of TF isoforms to 11 996 (Figure S13a). Notably, some families produced more isoforms under HS. For example, the IWGSC RefSeq v1.0 specifies 107 isoforms of HSFs, while 88 novel splicing isoforms of HSFs were identified from our data. Furthermore, in total, 422 and 111 wheat TFs were differentially expressed (DE‐TFs), and 191 and 179 genes were differentially spliced (DS‐TFs), including 20.9% and 15.3% DE‐TFs and 64.4% and 57.0% DS‐TFs identified as early HS‐responsive TFs (0–10 min after onset of HS), in leaves and grain, respectively, further supporting the rapid HS response (Tables S10 and S12).

**Figure 5:**
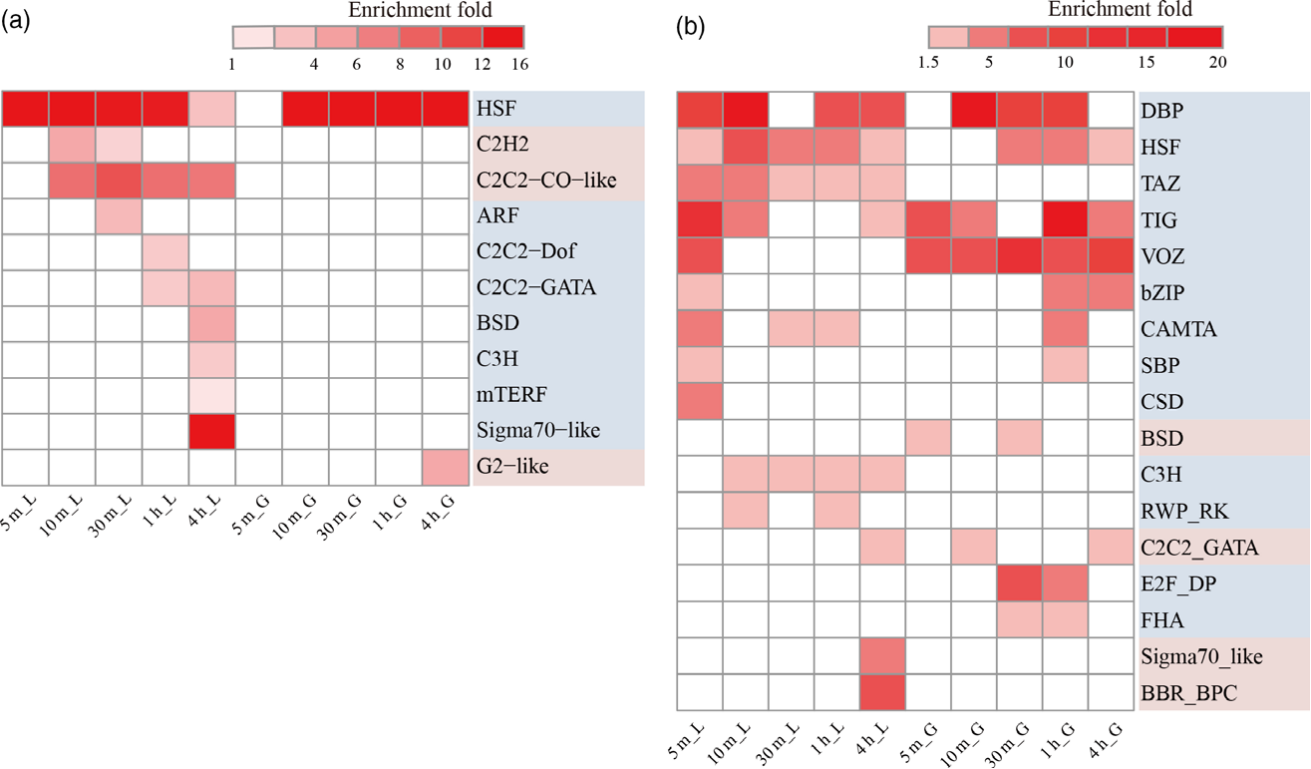
Differences in timing of diverse transcription factors (TFs) in the heat signaling and early heat response. (a, b) Heatmaps showing the fold enrichment of TFs in response to heat stress (HS) with transcriptional regulation (a) and alternative splicing regulation (b). Only significantly enriched families (*q *<* *0.05) are indicated. The *x*‐axis represents HS‐treated samples and the *y*‐axis represents the TF families. ‘L’ and ‘G’ in the sample names represent leaf and grain, respectively.

To investigate heat sensing and signaling in TFs, enrichment analysis was performed on heat‐responsive TFs at each time point. As expected, the HSF family was significantly enriched in DE‐TFs (Figure 5a). It is noteworthy that several members of the AP2, FAR1, MYB, bHLH, G3H, and NAC families were differentially expressed at the 10 min time point in grain, in parallel with the HSF response, providing evidence that these families participated in heat signaling transduction (Figure S13b). Consistent with this finding, the MYB family had previously been reported to activate HSF3 via direct protein−protein interactions in human proliferating cells, even under no HS (Kanei‐Ishii *et al*., 1997).

For AS regulation, the number of HS‐responsive TFs remained stable over time, as did the variation patterns observed for DSGs (Figures 5b and S13c). The TF families included DBP that exhibits both sequence‐specific DNA‐binding and protein phosphatase activity (Carrasco *et al*., 2005), TIG that is found in cell surface receptors (Bork *et al*., 1999), TAZ that is known as a novel CaM‐binding protein (Du and Poovaiah, 2004) and VOZ that involved in pollen development in Arabidopsis (Mitsuda *et al*., 2004), which were all over‐represented before or in parallel with HSF responses. Therefore, the TFs are extensively regulated by AS regulation in addition to the well known transcriptional regulation, especially in the early HS response.

### HSF‐independent and ‐dependent heat signaling and early heat responses

**Figure 6:**
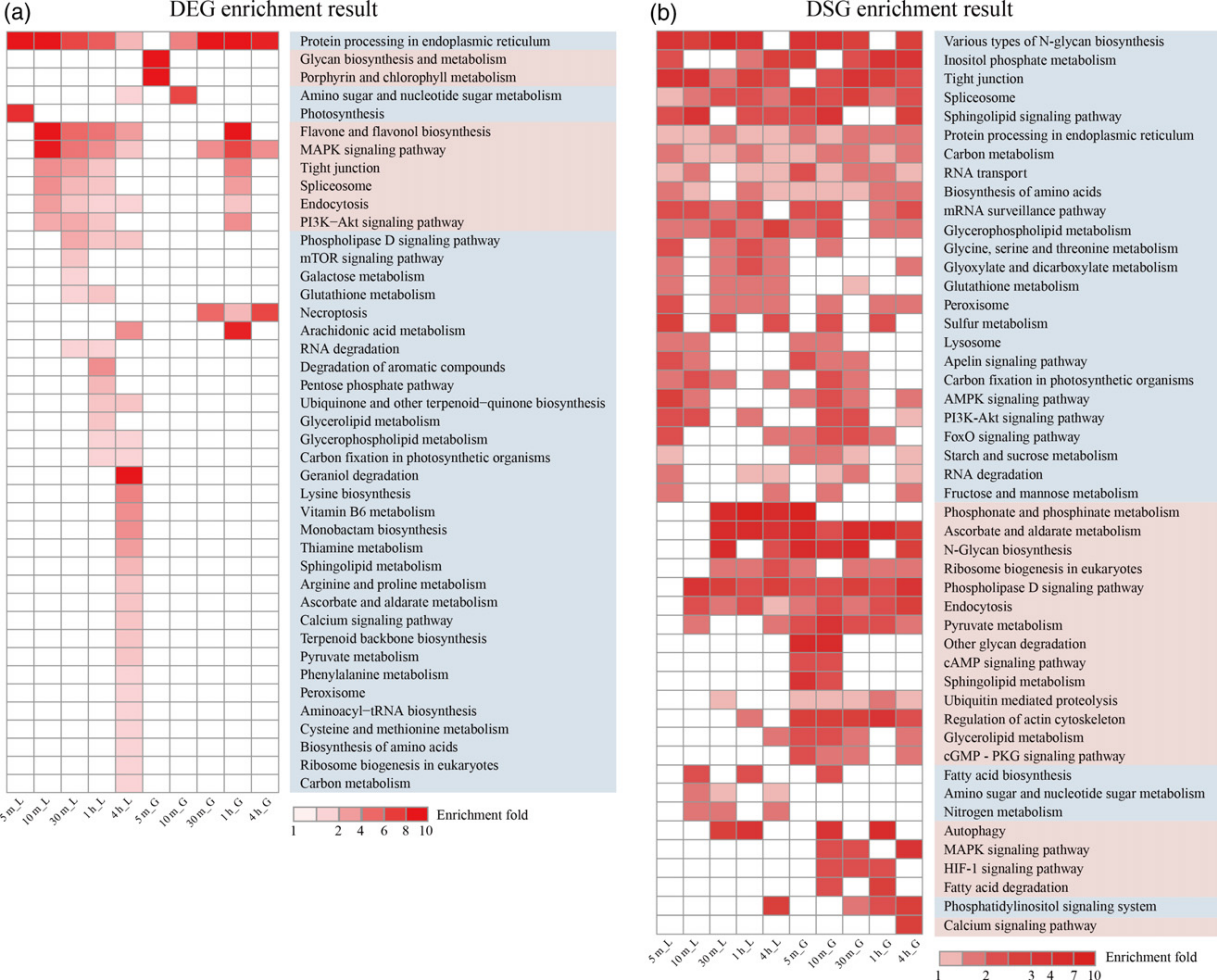
Heat signaling and early heat response processes. (a, b) Heatmaps showing the fold enrichment of enriched KEGG pathways for differentially expressed genes (DEGs) (a) and differentially spliced genes (DSGs) (b). Only significantly enriched pathways (*q *<* *0.05) are indicated. The full list of enriched pathways is presented in Tables S16 and S18. The *x*‐axis represents HS treatment time points and the *y*‐axis represents enriched KEGG pathways. ‘L’ and ‘G’ in the sample names represent leaf and grain, respectively.

Our intensive time‐course transcriptomes in the early HS response allow us to identify heat signaling and early heat response processes. For example, the DEGs at 5 min in grain that were altered in expression before the HSF responses provided an opportunity to investigate the upstream links to HSFs in the heat signaling transduction chain and HSF‐independent heat signaling. This set of genes was mainly over‐represented in multiple metabolic processes, including ‘glycan biosynthesis and metabolism’, ‘porphyrin and chlorophyll metabolism’ and ‘negative regulation of metabolic process’, as well as ‘the response to external biotic stimulus’ and ‘the enzyme regulator activity’, suggesting key roles and independent responses with HSFs of these processes in heat sensing and signaling (Figure 6a and Table S16). The DEGs in short‐term HS responses (5 min in leaves and 10 min in grain) that were altered in parallel with HSF responses, revealed the significant over‐representation of genes involved in ‘protein processing in the endoplasmic reticulum’, ‘photosynthesis’, ‘electron transport chain’, ‘carbohydrate metabolic process’, ‘seed oilbody biogenesis and development’, ‘oxidation−reduction process’, ‘response to stress’ and ‘intrinsic component alternation of membrane’, implying that these pathways, like the HSFs, were involved in heat signaling transduction. Similarly, several metabolism pathways were also altered in parallel with HSF responses, further supporting the important roles of metabolic alterations in heat signaling transduction.

The time‐series analysis also showed that the above processes were involved in heat sensing and signaling, and further reveal the thermal conductivity and signaling duration (Figures S14 and S15 and Table S17). For example, cluster9 in leaves involved in heat sensing and signaling (including photosynthesis) was temporally upregulated and then continuously downregulated between 5 and 30 min, and cluster1 in grain (including the ‘protein processing in endoplasmic reticulum’ as heat signal) was continuously upregulated between 0 and 1 h and then remained stable at a higher expression levels at later time points.

For AS regulation, due to a larger number of DSGs at the 5 min time point in leaves and grain, many KEGG pathways and GO terms were already significantly enriched at this point, such as ‘protein processing in the endoplasmic reticulum’, ‘carbon metabolism’, ‘spliceosome’, ‘glycerophospholipid metabolism’, and ‘biosynthesis of amino acids’, among others (Figure 6b and Table S18). Noticeably, although a few overlapping DSGs were observed between leaves and grain (Figure 3d), the enrichment analysis of DSGs demonstrated that the heat‐affected pathways were highly conserved between these two organs. This finding was different from the enrichment results obtained with DEGs that showed that the HS response in leaves was more intense than in grain (Figure 6 and Tables S16, S18). In particular, some biological processes (e.g., ‘spliceosome’, ‘amino acid metabolism’, ‘carbohydrate metabolism’, ‘glycerophospholipid metabolism’, ‘pyruvate metabolism’, ‘phospholipase D and PI3K‐Akt signaling pathway’, and ‘positive regulation of oxidoreductase activity’), which showed significant alterations in leaves but no alterations or less significant changes in grain in terms of transcriptional regulation, showed significant enrichment in these two organs, or predominantly in grain, in terms of AS regulation (Figure 6).

### Transcriptional and AS regulation modulate different genes and pathways in heat signaling and heat adaptation

**Figure 7:**
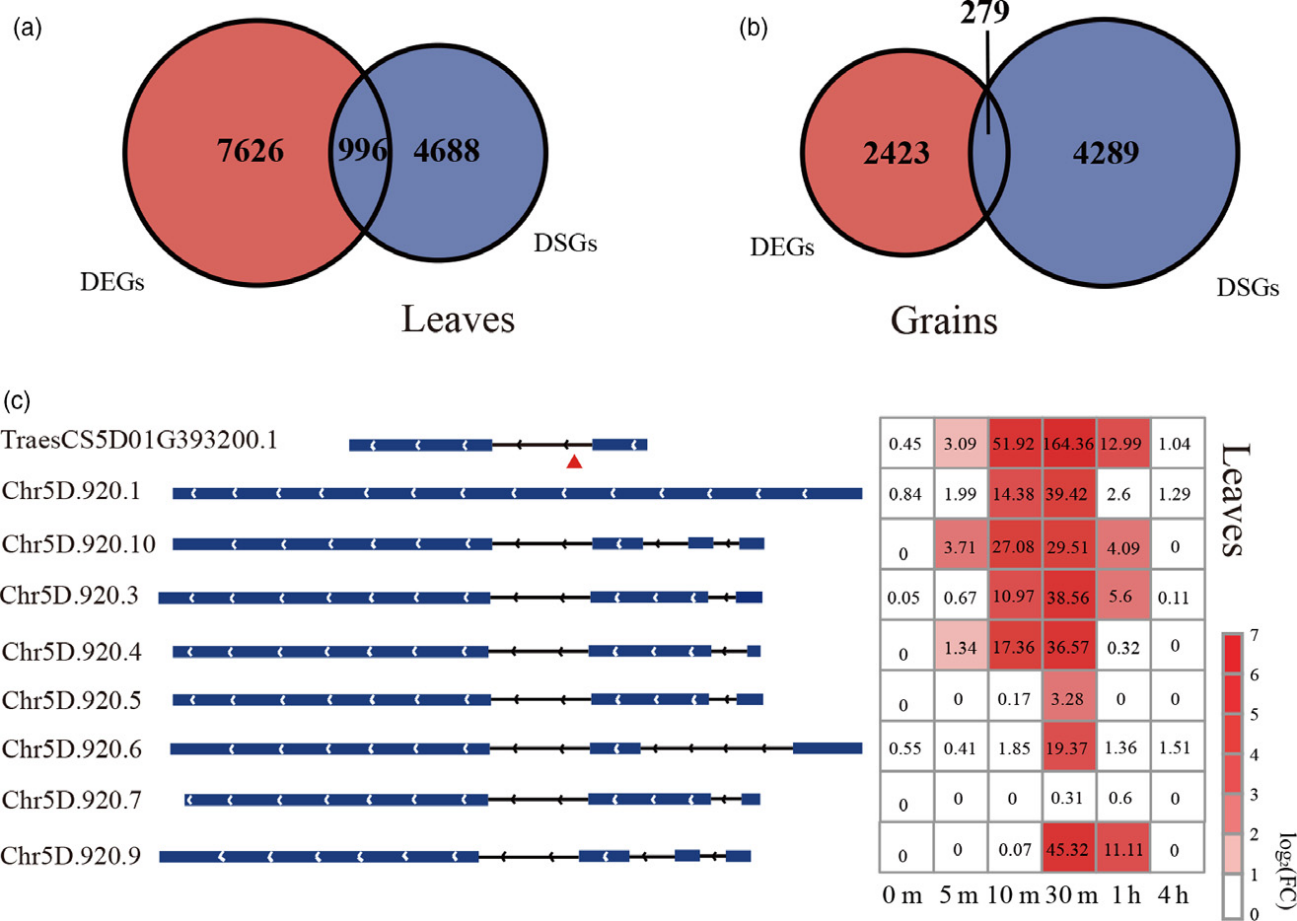
Overlap of differentially expressed genes (DEGs) and differentially spliced genes (DSGs), and a heat shock factors coding gene that responds to heat stress with both transcriptional regulation and alternative splicing regulation. (a) Overlap of DEGs and DSGs in leaves. (b) Overlap of DEGs and DSGs in grain. (c) Schematic representation of the isoforms produced by *TraesCS5D01G393200* (a member of the HSFA2 subfamily). Exons are represented as blue boxes and introns as lines. The isoforms whose names start with ‘Chr’ were novel isoforms identified from the PacBio data. The red triangle indicates the location of an in‐frame premature termination codon in the intron. For the right part, the numbers in rectangles represent average FPKM values of three replicates at each time point in leaves. The heatmap shows the fold change of each isoform at different time points (0 min time point was used as a control).

Using extensive HS‐response transcriptomes and a hybrid sequencing strategy, we had an unprecedented opportunity to investigate the relationship between transcriptional and AS regulation, which has always been an intriguing question. We first compared DEGs with DSGs (Figures 7a,b and S16). Only 996 genes in leaves (11.55% of DEGs and 17.52% of DSGs) and 279 genes in grain (10.32% of DEGs and 6.11% of DSGs) overlapped between DEGs and DSGs, suggesting that transcriptional and AS regulation modulate different target genes in the HS response. To further investigate whether the specific genes derived from different members of one homologous gene set, we searched the DSG‐specific genes for homologues of DEG‐specific genes and *vice versa*. An average of 14.81% of DEG‐specific genes and 4.27% of DSG‐specific genes possessed gene homologues in the opposite group in grain, and the relative numbers in leaves were 12.20% and 16.26%. These results showed that the target genes of transcriptional regulation and AS regulation were truly different, especially in the early HS response (Figure S16).

To further understand the difference between AS and transcriptional regulation, we performed GO and KEGG enrichment analysis for DEG‐specific and DSG‐specific genes, and demonstrated that they affected different pathways (Table S19). For example, ‘protein processing in endoplasmic reticulum’ and ‘MAPK signaling pathway’ were only enriched for DEG‐specific genes, while ‘ascorbate and aldarate metabolism’ was only enriched for DSG‐specific genes.

The overlapping genes between DEGs and DSGs were regulated both transcriptionally and post‐transcriptionally and may modulate critical functions in the HS response. The significantly over‐represented categories of this gene set mainly related to ‘response to temperature stimulus’ and ‘protein processing in endoplasmic reticulum’ (Table S19), which were well recognized to be involved in the HS response. We further mapped the DEGs and DSGs to these jointly regulated pathways and found that their distribution was scattered rather than being clustered to specific parts of these pathways (Figures S17 and S18). Furthermore, many HSF‐ and HSP‐encoding genes expectedly were contained in this gene set. For example, *TraesCS5D01G393200* (a member of the HSFA2 subfamily) was significantly upregulated and was also differentially spliced under HS (Figure 7c). Interestingly, the isoform Chr5D.920.1 was generated by the splicing of an intron of *TraesCS5D01G393200* and may produce a polypeptide with an incomplete DNA‐binding domain of HSF due to an in‐frame premature termination codon in the retained intron. This AS pattern was similar to that reported for the truncated isoforms of *HSFA2‐II* and *HSFA2‐III* in Arabidopsis, which was involved in the response to moderate and severe HS, respectively (Sugio *et al*., 2009; Liu *et al*., 2013).

### Differential responses and partitioned functions among three subgenomes in wheat heat sensing and signaling

**Figure 8:**
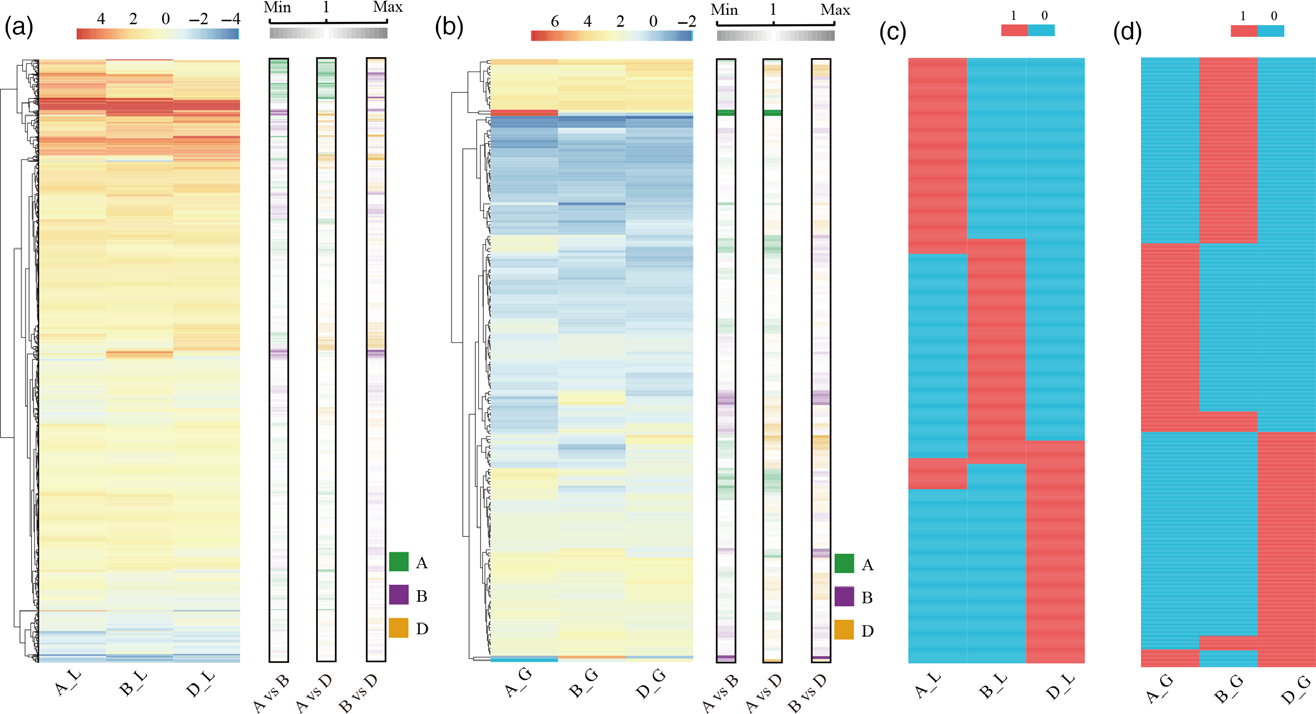
Subgenome bias in the heat stress response. (a, b) Heatmaps showing the fold change in the expression of each gene in each homologous triplet at the 30 min time point in leaves (a) and at the 1 h time point in grain (b). Only the triplets that contain differentially expressed genes are displayed. For the symbols on the *x*‐axis, ‘A’, ‘B’ and ‘D’ represent the A‐, B‐ and D‐homeologues in the triplets, respectively. ‘L’ and ‘G’ represent the leaves and grain, respectively. In the right part, the heatmaps display the pairwise ratio of the fold changes between the A‐, B‐ and D‐homeologues. The green, purple and orange represent the different responses of the A‐, B‐ and D‐homeologues in each pairwise comparison. A versus B: the comparison between the A‐ and B‐homeologues, A versus D: the comparison between the A‐ and D‐homeologues, and B *versus* D: the comparison between the B‐ and D‐homeologues. (c, d) Heatmaps display the differentially spliced genes (DSGs) in each homologous triplet at the 30 min time point in leaves (c) and at the 1 h time point in grain (d). Only the triplets that contain DSGs are displayed. The symbol ‘1’ indicates that the gene is a DSG and the symbol ‘0’ indicates that the gene is not a DSG.

As an allohexaploid species, wheat contains three highly similar and redundant subgenomes and the subgenomic bias was observed for several aspects of the development and stress response in wheat (Pfeifer *et al*., 2014; Liu *et al*., 2015; Nussbaumer *et al*., 2015; Powell *et al*., 2017). First, we calculated the number of DEGs and DSGs in each subgenome, showing that the B subgenome contained the most DEGs and DSGs (Figure S19). Then, to further understand the subgenomic bias, we identified 7287 homologous triplets, that is genes that were homologous between the subgenomes and had only one copy present in each of the subgenomes (Table S20). Of these, 202 (2.77%) and 1285 (17.63%) triplets in grain and 1131 (15.52%) and 1306 (17.92%) triplets in leaves contained DEGs and DSGs, respectively. Among the triplets that contained DEGs, by calculating the ratio of the fold change between the A‐, B‐, and D‐homeologues, 172 triplets in grain (85.15%) and 866 triplets in leaves (76.57%) exhibited differential HS responses under at least one time point according to the criteria of a 1.5‐fold change (Figure 8a,b). Unexpectedly, all of the DSG‐containing triplets in grain and leaves were found to possess only one or two homologues that were differentially spliced at each time point, suggesting that differential AS responses occurred in all triplets (Figure 8c,d).

Then, we classified the triplets into distinct categories based on the differential HS responses between the A‐, B‐, and D‐homeologues (Figures S20 and S21). Interestingly, during the HS response, homologues were differentiated based on specific biological pathways (Table S21), implying the partitioned functions among subgenomes in the HS response. For example, the category, in which the A‐ homeologues were more highly upregulated than the B‐ and D‐homeologues in grain at the 10 min time point, revealed the significant over‐representation of genes involved in protein processing in the endoplasmic reticulum. The category, in which only the D‐homeologues were differentially spliced at the 5 min time point in grain, contained the genes involved in the spliceosome. Collectively, the three subgenomes of wheat exhibited differential responses and partitioned functions in the HS response.

## Discussion

### Hybrid sequencing is an effective approach for excavating genomic diversity and plasticity of polyploidy species

Using hybrid sequencing technology, three key findings that contribute to our understanding of the current wheat genome were as follows. First, 4947 gene loci were newly discovered on the IWGSC RefSeq v1.0; second, 70 285 isoforms were identified that were absent from the IWGSC RefSeq v1.0 annotation and were defined as novel isoforms; and third, hybrid sequences significantly improved the length of the high‐quality wheat transcript. These findings highlighted the hybrid sequencing strategy as an effective compensatory strategy for the existing annotation of IWGSC RefSeq v1.0. These newly discovered genes and isoforms provide valuable insight for future research on wheat gene clones, as well as functional studies and studies of genome evolution.

The erroneous correction of PacBio reads with Illumina short reads was a critical step in the hybrid sequencing strategy, and several tools have been developed for this purpose (Hackl *et al*., 2014; Rhoads and Au, 2015). However, the existence of highly homologous subgenomes in wheat makes this step more challenging. For example, the PacBio reads (originating from the A gene in the A subgenome) could be corrected not only by the Illumina short reads that originated from the A gene, but also by the Illumina short reads that originated from the homologous genes of the A gene in the B or D subgenomes. Two PID measures (Figure S1) were introduced into our analysis to reduce the adverse effects of highly homologous subgenomes in wheat, by making full use of the single nucleotide polymorphisms between homologous genes in the same or different subgenomes and comparing the mapping scores before and after erroneous correction.

To evaluate the effectiveness and accuracy of the PID strategy, we calculated the number of FLNC reads that mapped to another subgenome and had a higher PID after erroneous correction. Of the 705 335 FLNC reads, 3656 (0.52%) fitted these criteria and were involved in the identification of 2403 isoforms and 2242 gene loci. Further analysis showed that 734 isoforms and 616 gene loci were defined only by the above 3656 FLNC reads and were not supported by other FLNC reads. Of these, 290 isoforms and 610 gene loci were also annotated in the IWGSC RefSeq v1.0. Therefore, the identification of 444 (0.45%) isoforms and three (0.006%) gene loci may be affected by the homologous subgenomes. These evaluation results showed that the PID strategy would be an effective strategy for erroneous correction and could be applied in future hybrid sequencing studies, especially for polyploidy species.

### The different patterns between transcriptional regulation and AS regulation

Previous studies have proven that transcriptional regulation and AS regulation are two parallel processes that function independently in the stress response with little overlap between target genes (Li *et al*., 2013; Chang *et al*., 2014; Ding *et al*., 2014; Ling *et al*., 2015; Liu *et al*., 2018). In our analysis, we confirmed that transcriptional and AS regulation also affect distinct gene sets and biological processes, providing further evidence for their independent regulatory roles.

Notably, the large number of DSGs at the 5 min time point in leaves and grain suggest that AS may participate in early heat sensing. It remains unclear how AS senses the heat signal and whether transcriptional regulation is involved in this sensing process. In our results, the AS response occurred much earlier than the HSF response, suggesting that the AS response is not transduced from HSFs. Whereas, it was confirmed that AS participates in transcriptional regulation in view of the broad AS events that occurred in many HSFs and TFs under HS. Taken together, our results demonstrate that AS regulation is independent or partially independent of transcriptional regulation and played an important role in the early heat response, providing valuable insight for future heat sensing and signaling studies.

### Time scales for heat sensing and signaling

Our results showed that the heat responses of wheat flag leaves and grain, the two most important organs in wheat grain‐filling processes, were distinct in terms of HS‐response times, HS‐responsive genes, and HS‐affected pathways. HS responses in leaves were more severe than those in grain, and grain showed some degree of heat resistance. This finding was consistent with our previous observation that sHSPs, as key genes in the HS response, maintain higher basic transcriptional levels in grain than in leaves under normal temperatures (Wang *et al*., 2017b). To understand whether different organs employ different strategies in the HS response, we comprehensively compared the heat response in flag leaves and grain, highlighting the differences among organs in response to the same stress and suggesting that different strategies and approaches should be considered in mechanistic studies and thermotolerance improvement practices for different organs in the future.

The different time courses for the heat response of leaves and grain provide opportunities to evaluate heat sensing and signaling. In this study, the DEGs involved in protein processing in the endoplasmic reticulum were significantly enriched after 5 min HS treatment in leaves and 10 min in grain, strongly supporting the hypothesis that the HSP competition model plays a primary role in early HS sensing and signaling (Nollen and Morimoto, 2002). Similarly, we also found that plant hormone signals and MAPK signaling altered after the response time of HSFs, suggesting that the signaling of HSF responses is not transduced from the MAPK pathway (Campbell *et al*., 2001; Wang and Li, 2006; Wahid *et al*., 2007). Metabolic alterations are usually regarded as a consequence of heat adaptations (Mangelsen *et al*., 2011); however, the carbon metabolism‐related pathway and metabolism of some amino acids and lipids were significantly over‐represented in the earliest HS responses, prior to and/or in parallel with HSF responses, indicating that metabolism may be one of the important links in the heat sensing and signaling transduction chain. These findings provide critical clues for further dissecting the complex heat response gene network.

### The evolution of polyploidy wheat under HS

As an allohexaploid, bread wheat contains three highly homologous subgenomes and a highly redundant gene set. Interestingly, different homologues were exploited by different organs and different regulatory systems in response to HS. The large number of homologous genes in allohexaploid wheat may provide more options for regulatory systems and organs, potentially contributing to the increased adaptability to different growth environments and resistance to stress compared with its ancestors (Dubcovsky and Dvorak, 2007).

After polyploidization events, homologous genes from the same ancestor have adjusted their expression in response to the environment, leading to potential functional divergence (Feldman and Levy, 2009). In this study, most of the triplets involved in the HS response exhibited different response patterns among homologous genes, especially in AS regulation, strongly suggesting that triplets in the wheat genome experience general functional divergence in the HS response. However, most HSFs and HSPs maintain similar heat response profiles among subgenomes, indicating little functional divergence occurred among the homologous genes of HSFs and HSPs. Based on this, we propose that different gene sets are subjected to different selection pressures and undergo different degrees of functional divergence in environmental adaptations. These results provide a perspective on the functional divergence between homologous genes induced by environmental stress, which may contribute to our understanding of genomic evolution.

In conclusion, we illustrate the spatio‐temporal landscape of heat adaptations in wheat filling grain and flag leaves at isoform resolution by combining second‐ and third‐generation sequencing, providing the most comprehensive heat‐responsive transcripts to date and complementing the recently released wheat reference genome. This integrated analysis uncovered the unexpectedly rapid process of heat sensing and signaling and suggested several pathways of HS sensing or early HS signaling, updating and improving previous assumptions in this area. Differential responses and partitioned functions between organs and subgenomes were observed, highlighting the evolutionary divergence and advantages of the polyploid nature of wheat in environmental adaptations. Our results provided comprehensive data for dissecting molecular mechanisms of early HS responses in wheat and clues for wheat thermotolerance improvement.

## Experimental procedures

### Plant materials

Wheat plants (*T. aestivum* cv. Chinese Spring) were first grown in a greenhouse under normal conditions. The plants were labeled when they began flowering. Then, the plants were transferred to growth chambers with a temperature regime of 24/17°C on a (14/10 h) day/night cycle, 12 days after anthesis. After 3 day/night cycles, the temperature was increased to 37°C after 4 h of light at 15 days after anthesis. The filling grain and flag leaves were harvested at 0 min, 5 min, 10 min, 30 min, 1 h and 4 h under HS (Figure 1a,b) and were immediately frozen in liquid N_2_ for the subsequent isolation of RNA. The RNAs of 36 samples (six time points for each of the two organs, three biological replicates per time point) were subjected to 150 bp paired‐end sequencing using the Illumina HiSeq X Ten platform, and then the RNAs of 18 samples from each organ were mixed in equal volume and sequenced on the PacBio RS II platform.

### Illumina RNA‐Seq library construction

In total, 3 μg RNA per sample was used as the input material and mRNA was purified from the total RNA using poly‐T oligo‐attached magnetic beads. Sequencing libraries were generated using the NEBNext^®^ Ultra™ RNA Library Prep Kit for Illumina^®^ (NEB, USA) following the manufacturer's recommendations and index codes were added to attribute sequences to each sample. The library quality was assessed on the Agilent Bioanalyzer 2100 system.

### PacBio sequencing library construction

The cDNAs were prepared using the SMARTer PCR cDNA Synthesis Kit (Clontech) from 1 μg of purified polyA(+) RNA. cDNAs were then size selected (0.5–1, 1–2, 2–3, and 3–6 kb) using the BluePippin™ system (Sage Science). Then, Iso‐seq libraries were constructed according to the supplied protocol (http://www.pacb.com/wp-content/uploads/2015/09/Procedure-Checklist-Isoform-Sequencing-Iso-Seq-using-the-Clontech-SMARTer-PCR-cDNA-Synthesis-Kit-and-the-BluePippin-Size-Selection-System.pdf). For each of the four fraction size libraries, three single‐molecule real‐time (SMRT) cells were sequenced using P6‐C4 reagent on the PacBio RS II platform with 4 h sequencing movies for the leaf and grain tissues, respectively.

### PacBio data analysis

PacBio raw data were pre‐processed using the SMRT Pipe analysis workflow of the PacBio SMRT Analysis software suite (http://www.pacb.com/products-and-services/analytical-software/smrt-analysis/). Briefly, raw polymerase reads were filtered and trimmed to generate the subreads and read of inserts (ROIs) using the RS_Subreads protocol, requiring a minimum polymerase read length of 50 bp, a minimum polymerase read quality of 0.75, a minimum subread length of 50 bp, and a minimum of one full pass. FLNC reads were regarded as those containing a 5ʹ adapter, 3ʹ adapter, and poly(A) tail in the expected arrangement with no additional copies of the adapter sequence within the ROI.

Error correction of FLNC reads with the high‐quality Illumina short reads was performed using Proovread version 2.12 (Hackl *et al*., 2014) with the default parameters. The quality of Illumina short reads was examined using FastQC (v0.11.5; http://www.bioinformatics.babraham.ac.uk/projects/fastqc). Sequencing adaptors and low‐quality bases in short reads were trimmed before the error correction of FLNC reads. FLNC reads before and after error correction were respectively mapped to the IWGSC RefSeq v1.0 using GMAP (version 2016‐09‐14; https://github.com/juliangehring/GMAP-GSNAP) (Wu and Watanabe, 2005) with ‘–no‐chimeras and ‐n 100’ parameters. To avoid additional errors introduced by the error correction process, we introduced two percentage‐of‐identity (PID)‐related measures (Figure S1), local PID, and global PID, to reduce the adverse effects of highly homologous subgenomes of wheat on FLNC reads mapping. The PID was calculated based on the ratio of the number of nucleotide matches between the pairs in the alignment for FLNC reads and wheat genome sequences. For each of the FLNC reads, we compared the PID of FLNC reads before and after error correction and retained the one with the highest PID. Only mapped reads with high mapping quality (global PID ≥ 95% and local PID ≥ 97%) and the highest PID value from only one genome location were defined as uniquely mapped reads and retained for further analysis.

### Identification of gene loci and isoforms

According to the read‐genome alignments, FLNC reads with the same splicing junctions were collapsed into one isoform. The isoforms that had a shorter 5′ terminal region but shared the introns and splicing sites in the remaining region compared with other isoforms, were regarded as transcripts degraded at the 5′ terminal region and were filtered out. For the remaining isoforms, the supporting evidence was examined. We retained isoforms supported with at least two FLNC reads, or one FLNC read with PID higher than 99%, or all junction sites that were fully supported by Illumina reads or annotations of the IWGSC RefSeq v1.0. Isoforms that overlapped by at least 20% of their length on the same strand were considered to be from the same gene locus. Newly discovered loci and isoforms were identified by comparing the identified loci and isoforms with the reference genome annotation using the same criterion as for loci and isoform identification. AS events were classified and characterized by comparing different isoforms of the same gene loci using Asprofile (Florea *et al*., 2013). Tissue‐specific gene loci and isoforms were determined by tracing the tissue of origin of FLNC reads, which was then used to identify the relative gene loci and isoforms.

### Expression abundance of genes and isoforms

For each of the 36 samples, the trimmed short reads were mapped to the wheat reference genome (IWGSC RefSeq v1.0) using TopHat (v2.1.1; https://ccb.jhu.edu/software/tophat) (Trapnell *et al*., 2009). RSEM (v1.3.0; https://deweylab.github.io/RSEM) (Li and Dewey, 2011) was used to calculate the isoform‐level expression in terms of FPKM and TPM (transcripts per million).

### Identification of differentially expressed genes and differentially spliced genes

To carry out differential expression analysis, transcript quantification results generated by RESM were processed and refined in successive steps. First, transcript and gene read counts were generated from TPM data correcting for possible gene length variations across samples that mainly derived from differential transcript usage, using the tximport 1.10.0 R package with the option ‘lengthScaledTPM’ (Soneson *et al*., 2015). Second, the corrected read count data of genes were used to estimate their expression in terms of FPKM. Third, the samples from the 0 min time point that were not treated by HS were used as the control, the corrected read count data of genes were imported into the R package EdgeR (v3.20.3; http://bioconductor.org/packages/release/bioc/html/edgeR.html) (Robinson *et al*., 2010) to identify DEGs with the criteria of a fold change ≥2.0, a false discovery rate (FDR)‐adjusted *P*‐value <0.05, and expression (FPKM ≥ 1) in at least one sample for each comparison.

Differentially expressed genes between leaves and grain under each HS time point were identified using EdgeR (Robinson *et al*., 2010), with double factor differential expression analysis (Li˗L0)˗(Gi˗G0), where ‘i’ represents a time point, ‘0’ represents the 0 min time point, ‘L’ represents leaf, and ‘G’ represents grain. A gene was regarded as a DEG if it showed a fold change ≥2, an FDR‐adjusted *P*‐value ≤0.05 and it was expressed (FPKM ≥ 1) in at least one sample for each comparison.

The HS‐responsive genes with AS regulation were defined based on the following criteria: (i) the highly expressed transcript set (FPKM ≥ 1.0 and all junction sites that were fully supported by Illumina reads) differed between the control and HS‐treated samples; (ii) for the highly expressed transcript (FPKM ≥ 1.0), the transcript expression percentage (transcript expression/the expression sum of all transcripts of the same gene) changed by more than 30% between the control and HS‐treated samples (Liu *et al*., 2018), with supported Illumina read counts ≥5 for both samples, and the supported Illumina reads significantly differed (chi‐squared test, FDR‐corrected *P*‐value <0.05) between the two samples.

### Gene set enrichment analysis

The GO descriptions were obtained by BLAST and BLAST2GO searches (Conesa *et al*., 2005). KEGG pathway mapping was performed using Kobas (Xie *et al*., 2011). The statistical significance of the enrichment of GO and KEGG pathways was examined using the hypergeometric distribution test, followed by multiple‐test correction using the Benjamini–Hochberg method (Benjamini and Hochberg, 1995).

### Identification of the wheat homologous triplets and the transcription factors

The high‐confidence genes annotated in the IWGSC RefSeq v1.0 were grouped into 22 481 clusters using OrthoMCL (v2.0.9; http://orthomcl.org/orthomcl). A gene cluster was regarded as a homologous triplet candidate if it consisted of three genes corresponding to the A, B and D subgenomes. The homologous triplet candidates were further filtered based on the bidirectional‐best‐BLAST (BBH) gene pairs generated from an all‐against‐all BLAST search of all high‐confidence genes. Only when any two of the three members met the BBH criteria, were the corresponding homologous triplet candidates defined as gene triplets.

The TFs were identified based on the domains of known TFs in the plant transcription factor database PlnTFDB 3.0 (http://plntfdb.bio.uni-potsdam.de/v3.0/). The domains of the protein corresponding to the newly identified transcripts in our analysis and the annotated transcripts in the IWGSC RefSeq v1.0 (generated from both high‐confidence genes and low confidence genes) were searched against the included domains and excluded domains of each TF in the PlnTFDB database using the hmmsearch function of the HMMER software, and only proteins with exactly the same included domains and not with the excluded domains were regarded as TFs.

### The lncRNA identification

Newly identified isoforms with length ≥200 nt were first searched against NCBI's NR database using BLASTX with default parameters. Isoforms were regarded to have protein‐coding capacity if they had BLASTX hits with e‐value below 1E‐5 and were filtered out. The remaining transcripts was further evaluated by CPAT (v1.2.2; http://lilab.research.bcm.edu/cpat/) (Wang *et al*., 2013), which were trained using 3853 lncRNAs and 4000 randomly selected protein‐coding mRNAs of Arabidopsis obtained from the NONCODE database (v5; http://www.noncode.org/download.php) and TAIR database (release Araport11; http://www.arabidopsis.org/download/index-auto.jsp?dir=/download_files/Genes), respectively. Isoforms with CPC score ≥0.44 were also considered to have protein‐coding capacity. The threshold of the CPC score (0.44) was optimized by applying a 10‐fold cross‐validation approach, resulting to the generation of a balanced sensitivity and specificity (0.91) for CPAT.

## Accession numbers

The data reported in this article have been deposited in the NCBI SRA database under accession no. SRP128236 (https://www.ncbi.nlm.nih.gov/sra/SRP128236). The sequences and annotations of newly discovered loci and isoforms, and the expression level of genes and isoforms can be downloaded from the Zenodo (https://zenodo.org/) with the https://doi.org/10.5281/zenodo.2541477.

## Conflict of Interest

The authors declare no conflict of interest.

## Author contributions

CM and SX designed the experiments; XW, SC, DL, YC, YL, ZL, and XN performed analyses of the hybrid sequencing data; XS carried out the RT‐PCR; XW, CM and SX wrote the manuscript. WS and QS contributed to experiment design and manuscript revision.

## Supporting information


**Figure S1.** Visualization of local and global PID definitions.
**Figure S2.** Schematic of the seven groups of PacBio isoforms.
**Figure S3.** RT‐PCR validation of novel gene loci and novel AS isoforms identified by PacBio.
**Figure S4.** The GO and KEGG annotation of novel isoforms that have BLAST hits in the NR, GO or KEGG databases.
**Figure S5.** Comparison of DEGs among the five HS treatment time points in leaves and grain.
**Figure S6.** Differences in HS response between leaves and grain.
**Figure S7.** Comparison of DSGs among the five HS treatment time points in leaves and grain.
**Figure S8.** Venn diagram of DEGs (a) and DSGs (b) between leaves and grain at different time points.
**Figure S9.** Visualization and number of alternative splicing modes.
**Figure S10.** Distribution of the expression levels of the isoforms (FPKM ≥ 1.0) generated by the four AS modes in the DSGs.
**Figure S11.** The expression variation patterns of each member of each HSF subfamily.
**Figure S12.** The expression variation patterns of each member of the HSP20, HSP70, HSP90 and HSP100 families.
**Figure S13.** HS‐responsive TFs.
**Figure S14.** The thermal conductivity and signaling duration in leaves.
**Figure S15.** The thermal conductivity and signaling duration in grain.
**Figure S16.** Comparison of DEGs and DSGs at each HS treatment time point in leaves and grain.
**Figure S17.** Schematic representation of the protein processing in endoplasmic reticulum pathway exported from the KEGG database.
**Figure S18.** Schematic representation of the spliceosome pathway exported from the KEGG database.
**Figure S19.** Distribution of DEGs and DSGs among the three wheat subgenomes at each HS treatment time point.
**Figure S20.** Heatmaps display the DEGs in each homologous triplet at each time point.
**Figure S21.** Heatmaps display the DSGs in each homologous triplet at each time point.

**Table S1.** General properties of the reads produced by Illumina sequencing and PacBio sequencing.

**Table S2.** The alignment of FLNC reads with the IWGSC RefSeq v1.0.

**Table S3.** The gene and isoform annotations of the IWGSC RefSeq v1.0 and PacBio data.

**Table S4.** The genes and isoforms identified in the PacBio data.

**Table S5.** The BLAST hits of the newly discovered transcripts among the public databases.

**Table S6.** The domain annotation and enrichment of newly identified loci in InterPro database.

**Table S7.** The ratio of repeat sequences in newly discovered gene regions.

**Table S8.** The annotation of the newly discovered loci.

**Table S9.** The list of lncRNAs and their HS responses.

**Table S10.** The list of DEGs.

**Table S11.** The KEGG and GO enrichment analysis of genes which show different HS response between leaves and grain.

**Table S12.** The list of DSGs.

**Table S13.** The number and ratio of the four splicing modes in all PacBio isoforms or in the isoforms generated by DSGs.

**Table S14.** The classification of HSFs and their response to HS.

**Table S15.** The classification of HSPs and their response to HS.

**Table S16.** The KEGG and GO enrichment analysis of DEGs.

**Table S17.** The KEGG and GO enrichment analysis of DEG clusters in leaves and grain.

**Table S18.** The KEGG and GO enrichment analysis of DSGs.

**Table S19.** The KEGG and GO enrichment analysis of DEG‐ and DSG‐specific genes and overlapping genes between DEGs and DSGs.

**Table S20.** The list of identified homologous triplets.

**Table S21.** The KEGG and GO enrichment analysis of distinct categories based on the differential HS responses between the A‐, B‐ and D‐homeologues.
