# Supplementary material for: Hybrid sequencing reveals insight into heat sensing and signaling of bread wheat
Source: Plant J. 2019 Apr 23;98(6):1015–32. doi: 10.1111/tpj.14299 (PMC6850178; doi:10.1111/tpj.14299)
Supplement: Supplementary file 1 — Figure S1. Visualization of local and global PID definitions. Figure S2. Schematic of the seven groups of PacBio isoforms. Figure S3. RT‐PCR validation of novel gene loci and novel AS isoforms identified by PacBio. Figure S4. The GO and KEGG annotation of novel isoforms that have BLAST hits in the NR, GO or KEGG databases. Figure S5. Comparison of DEGs among the five HS treatment time points in leaves and grain. Figure S6. Differences in HS response between leaves and grain. Figure S7. Comparison of DSGs among the five HS treatment time points in leaves and grain. Figure S8. Venn diagram of DEGs (a) and DSGs (b) between leaves and grain at different time points. Figure S9. Visualization and number of alternative splicing modes. Figure S10. Distribution of the expression levels of the isoforms (FPKM ≥ 1.0) generated by the four AS modes in the DSGs. Figure S11. The expression variation patterns of each member of each HSF subfamily. Figure S12. The expression variation patterns of each member of the HSP20, HSP70, HSP90 and HSP100 families. Figure S13. HS‐responsive TFs. Figure S14. The thermal conductivity and signaling duration in leaves. Figure S15. The thermal conductivity and signaling duration in grain. Figure S16. Comparison of DEGs and DSGs at each HS treatment time point in leaves and grain. Figure S17. Schematic representation of the protein processing in endoplasmic reticulum pathway exported from the KEGG database. Figure S18. Schematic representation of the spliceosome pathway exported from the KEGG database. Figure S19. Distribution of DEGs and DSGs among the three wheat subgenomes at each HS treatment time point. Figure S20. Heatmaps display the DEGs in each homologous triplet at each time point. Figure S21. Heatmaps display the DSGs in each homologous triplet at each time point. [file TPJ-98-1015-s001.pdf]

Hybrid sequencing reveals insight into heat sensing and signaling of bread wheat

Xiaoming Wang[1], Siyuan Chen[2,3], Xue Shi[1], Danni Liu[4], Peng Zhao[1], Yunze Lu[1], Yanbing Cheng[4], Zhenshan Liu[1], Xiaojun Nie[1], Weining Song[1], Qixin Sun[5,1], Shengbao Xu[1*], Chuang Ma[2,3*]

[1]*State Key Laboratory of Crop Stress Biology for Arid Areas, College of Agronomy, Northwest A&F University, Yangling 712100, Shaanxi, China.*

[2]*State Key Laboratory of Crop Stress Biology for Arid Areas, College of Life Sciences, Northwest A&F University, Yangling 712100, Shaanxi, China.*

[3]*Center of Bioinformatics, College of Life Sciences, Northwest A&F University, Yangling 712100, Shaanxi, China.*

[4]*Frasergen, Wuhan East Lake High-tech Zone, Wuhan 430075, China.*

[5]*Department of Plant Genetics & Breeding, China Agricultural University, Yuanmingyuan Xi Road No. 2, Haidian District, Beijing 100193, China.*

***Correspondence:** Shengbao Xu, xushb@nwsuaf.edu.cn and Chuang Ma, chuangma2006@gmail.com

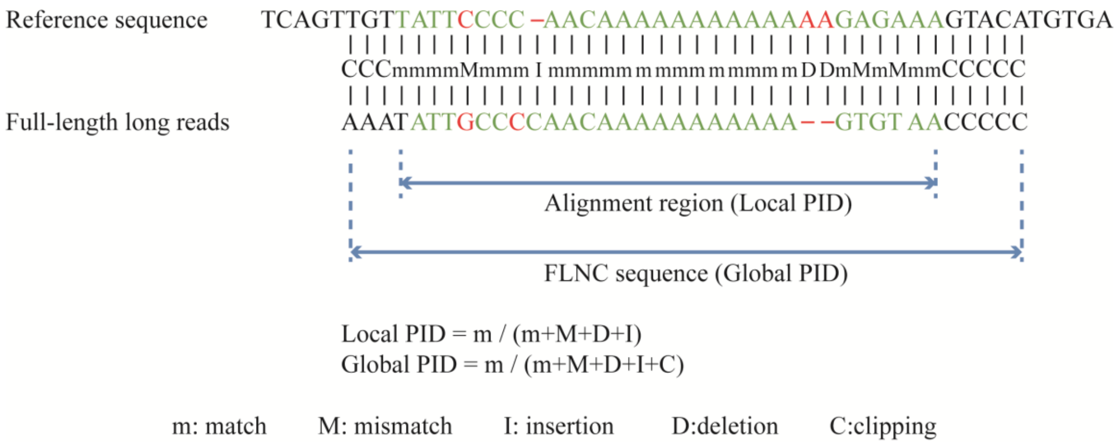

Supplemental Figure 1. Visualization of local and global PID definitions.

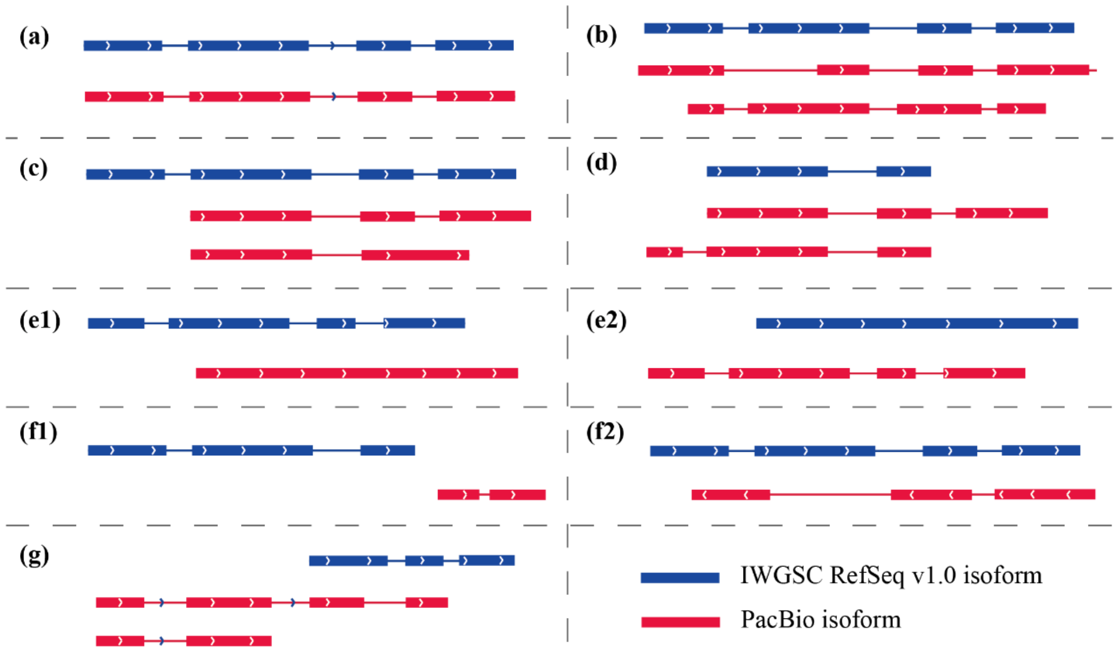

Supplemental Figure 2. Schematic of the seven groups of PacBio isoforms

(a) Isoforms have been annotated in the IWGSC RefSeq v1.0.

(b) Novel isoforms that contain multiple exons and have different splice sites and introns compared with annotated isoforms in the IWGSC RefSeq v1.0.

(c) Novel isoforms that contain multiple exons sequentially shared the introns of the annotated isoforms in the IWGSC RefSeq v1.0.

(d) Novel isoforms that contain multiple exons sequentially contained the introns of the annotated isoforms in the IWGSC RefSeq v1.0.

(e) Novel isoforms that contained a single exon and the annotated isoforms that contained multiple exons, or vice versa.

(f) Novel isoforms derived from novel gene loci.

(g) Novel isoforms that were non-overlapping with known isoforms in the same gene loci.

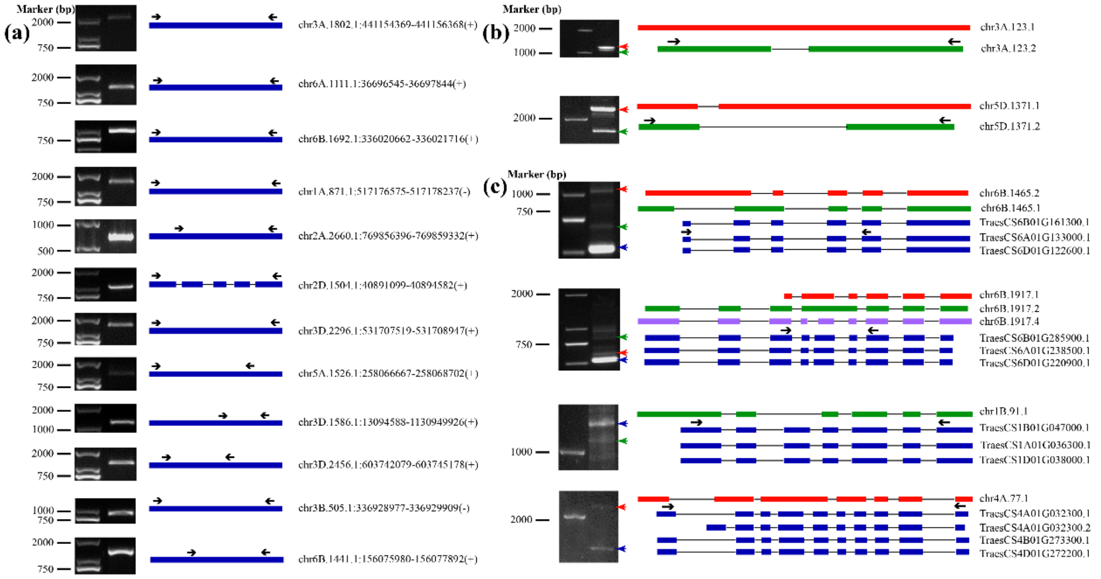

Supplemental Figure 3. RT-PCR validation of novel gene loci and novel AS isoforms identified by PacBio. The gel banding size and clone sequences of 14 selected novel genes, and the gel banding size of the novel isoforms were consistent with the corresponding genes and isoforms. (a) RT-PCR validation of novel gene loci. Mixed RNAs of grain and leaf samples of Chinese Spring wheat were used for PCR. The 14 novel genes (12 genes without AS and two genes with AS) were screened out from randomly selected novel genes based on whether specific primers could be designed. Forward and reverse primers are shown as arrows. A schematic representation of the genes is shown. Exons are represented as blue boxes and introns as lines. The locations of the genes on chromosomes are shown on the right. Plus and minus signs represent the forward and reverse strands, respectively. (b) RT-PCR validation of novel isoforms produced by novel gene loci. Exons are represented by filled boxes and introns by lines. Forward and reverse primers are shown as arrows. The novel isoforms are colour-coded and their PCR products are indicated by arrows with relative colours in the gel. (c) RT-PCR validation of novel isoforms produced by known gene loci in the IWGSC RefSeq v1.0. To reduce the adverse effects of highly homologous subgenomes, we firstly identified homologous triplets (i.e., homologous

genes between subgenomes that had only one copy present in each of the subgenomes) to avoid the adverse effects of highly homologous subgenomes, and then selected the triplets in which only one of the three genes produced novel isoforms. The gene models in the IWGSC RefSeq v1.0 are shown in blue and the novel isoforms identified in PacBio are shown in red/green/purple. Exons are represented by filled boxes and introns by lines. Forward and reverse primers are shown as arrows. The PCR products are indicated by arrows with relative colours in the gel.

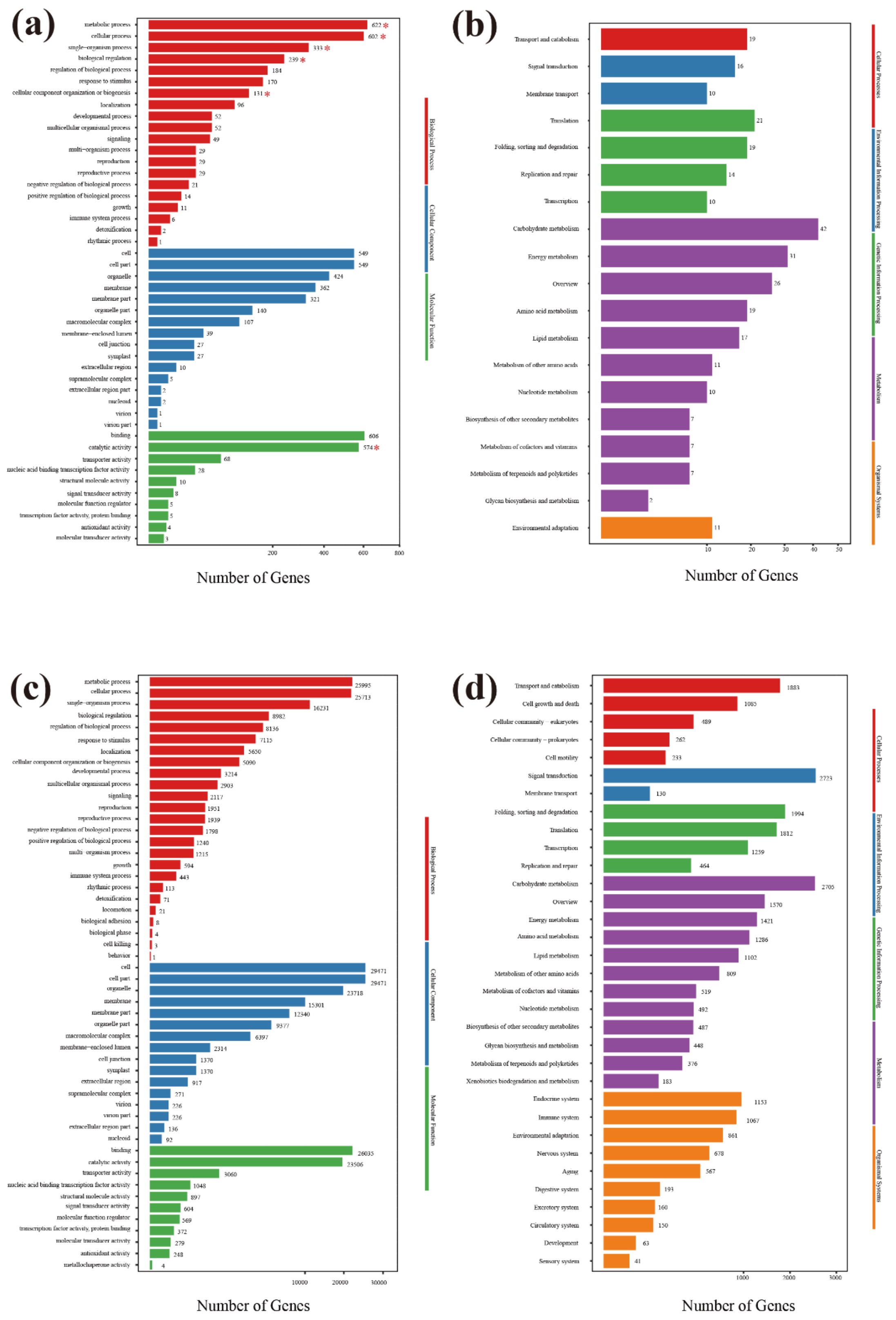

Supplemental Figure 4. The GO and KEGG annotation of novel isoforms that have BLAST hits in the NR, GO or KEGG databases

(a-b) The GO (a) and KEGG (b) annotations of novel isoforms from newly discovered gene loci. “*” indicates the GO term was significantly enriched in this data set.

(c-d) The GO (c) and KEGG (d) annotations of novel isoforms from annotated gene loci in IWGSC RefSeq v1.0.

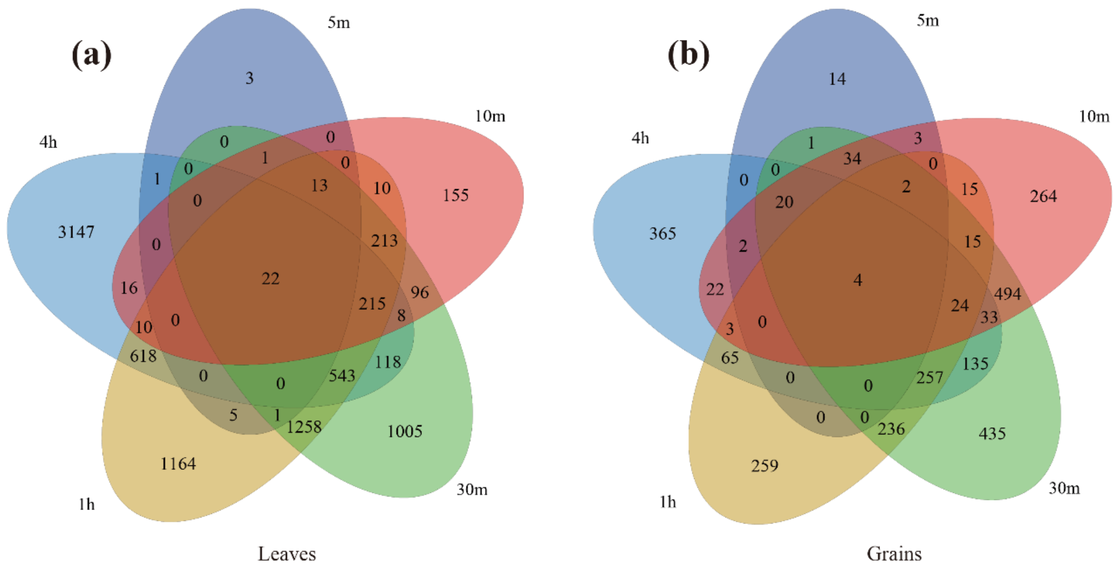

Supplemental Figure 5. Comparison of DEGs among the five HS treatment time points in leaves (a) and grains (b).

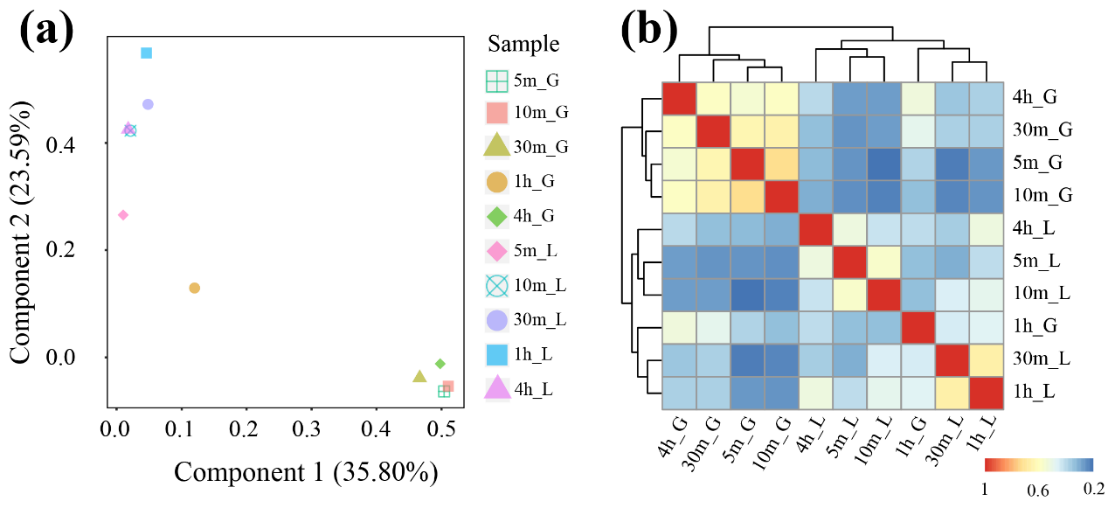

Supplemental Figure 6. Differences in HS response between leaves and grains. (a) The PCA of responsive transcriptomes. (b) The correlation of responsive transcriptomes for each HS time point. The FPKM value of each gene was normalized to the control and then subjected to PCA and correlation analysis. “L” and “G” in the sample names represent leaf and grain, respectively.

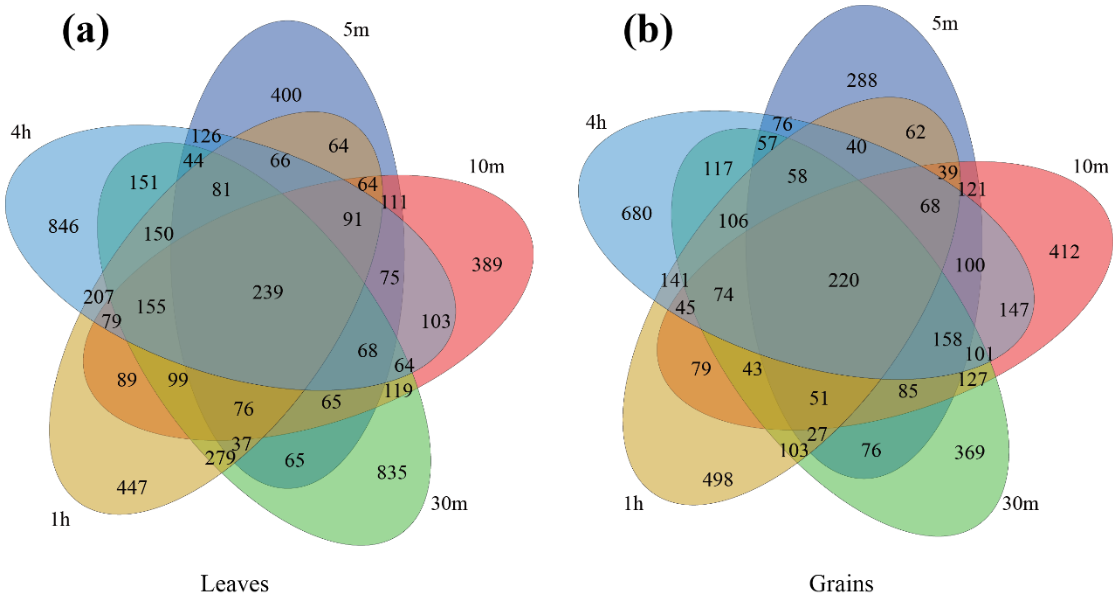

Supplemental Figure 7. Comparison of DSGs among the five HS treatment time points in leaves (a) and grains (b).

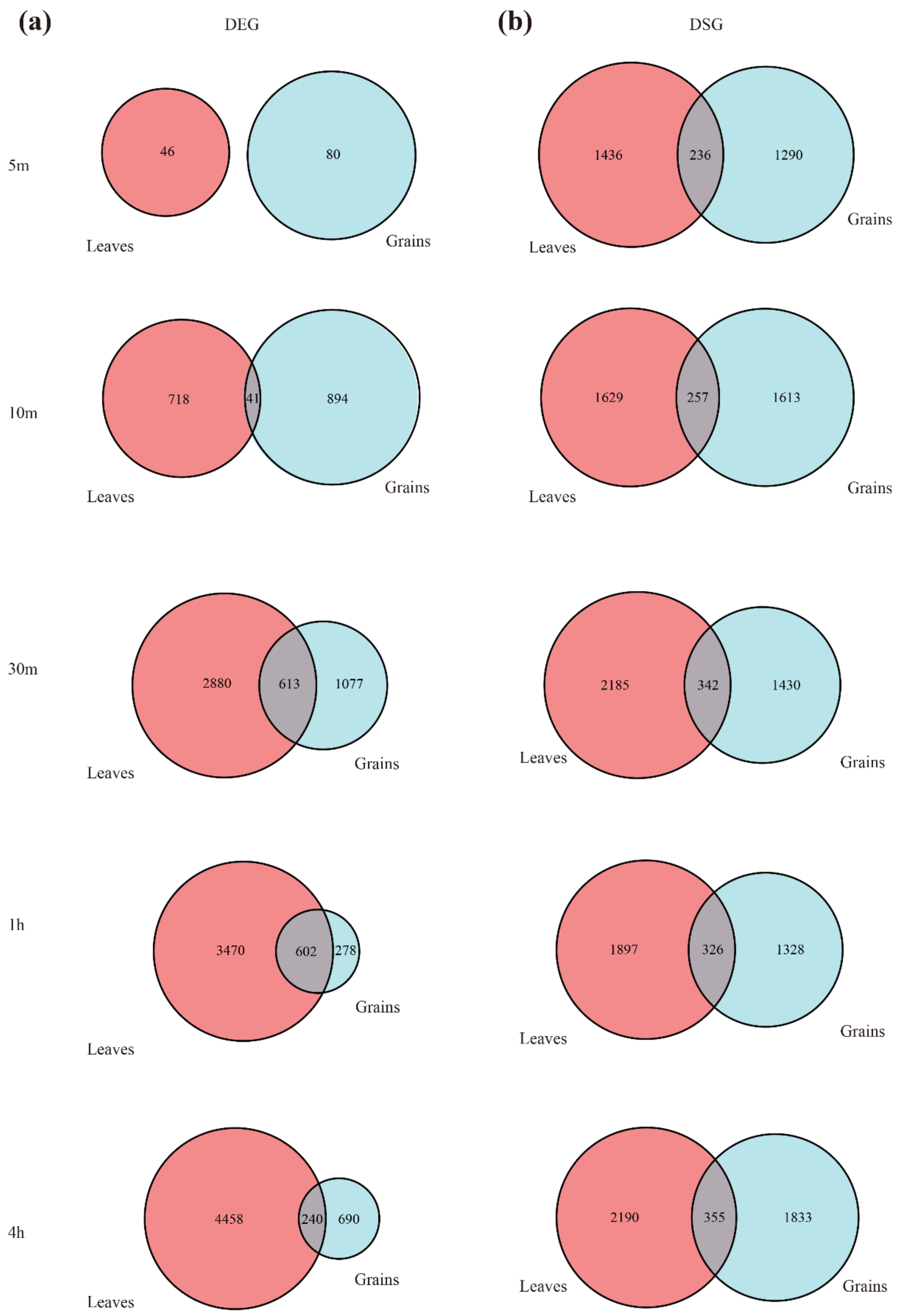

Supplemental Figure 8. Venn diagram of DEGs (a) and DSGs (b) between leaves and grains at different time points.

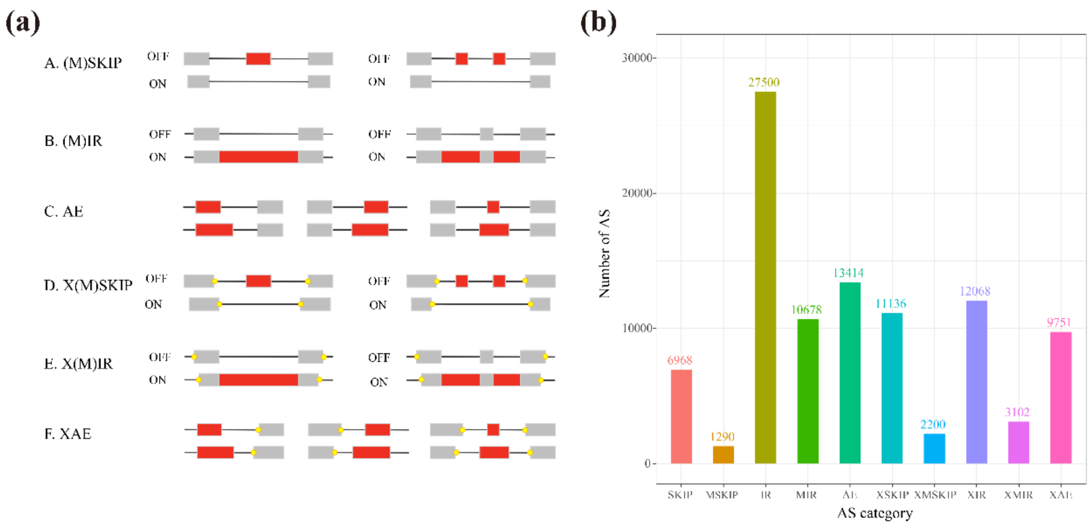

Supplemental Figure 9. Visualization and number of alternative splicing modes. (a) (A) Skipping of single exon (SKIP) and multiple exons (MSKIP). (B) Retention of single (IR) and multiple (MIR) introns. (C) Alternative exon ends (5′, 3′, or both) (AE). (D) Exon skipping and alternative exon ends simultaneously occurred in one isoform, and the yellow points represent alternative exon ends. (E) Intron retention and alternative exon ends simultaneously occurred in one isoform, and the yellow points represent alternative exon ends. (F) Multiple exons that had undergone AE in one isoform. (b) The number of each mode of alternative splicing.

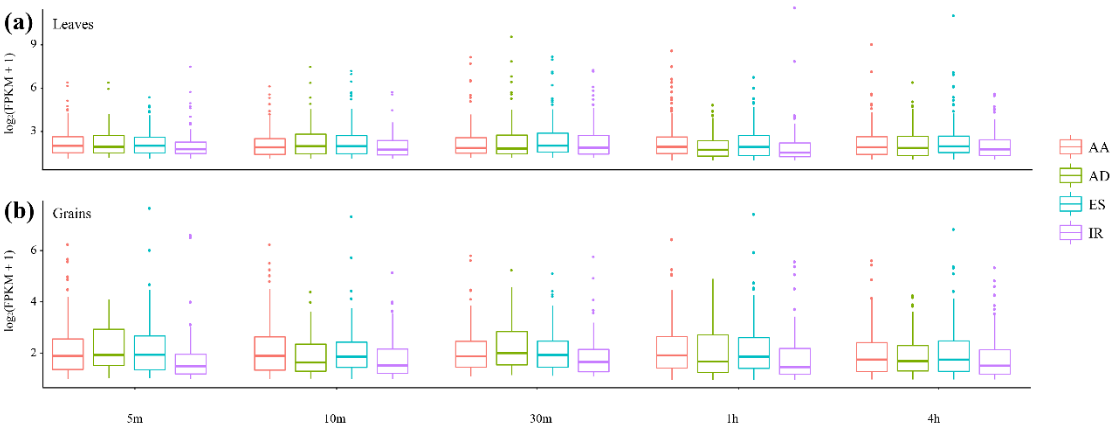

Supplemental Figure 10. Distribution of the expression levels of the isoforms (FPKM ≥ 1.0) generated by the four AS modes in the DSGs. (a-b) isoforms generated by DSGs in leaves (a) and in grains (b). The *x*-axis represents HS treatment time points and the *y*-axis represents the log2-transformed FPKM values. ES: exon skipping, IR: intron retention, AD: alternative donor sites, AA: alternative acceptor sites. Because the AS events in one isoform can consist of different AS types, isoforms that

exhibited only one of the four AS modes were analysed to clarify the different characteristics of the AS modes.

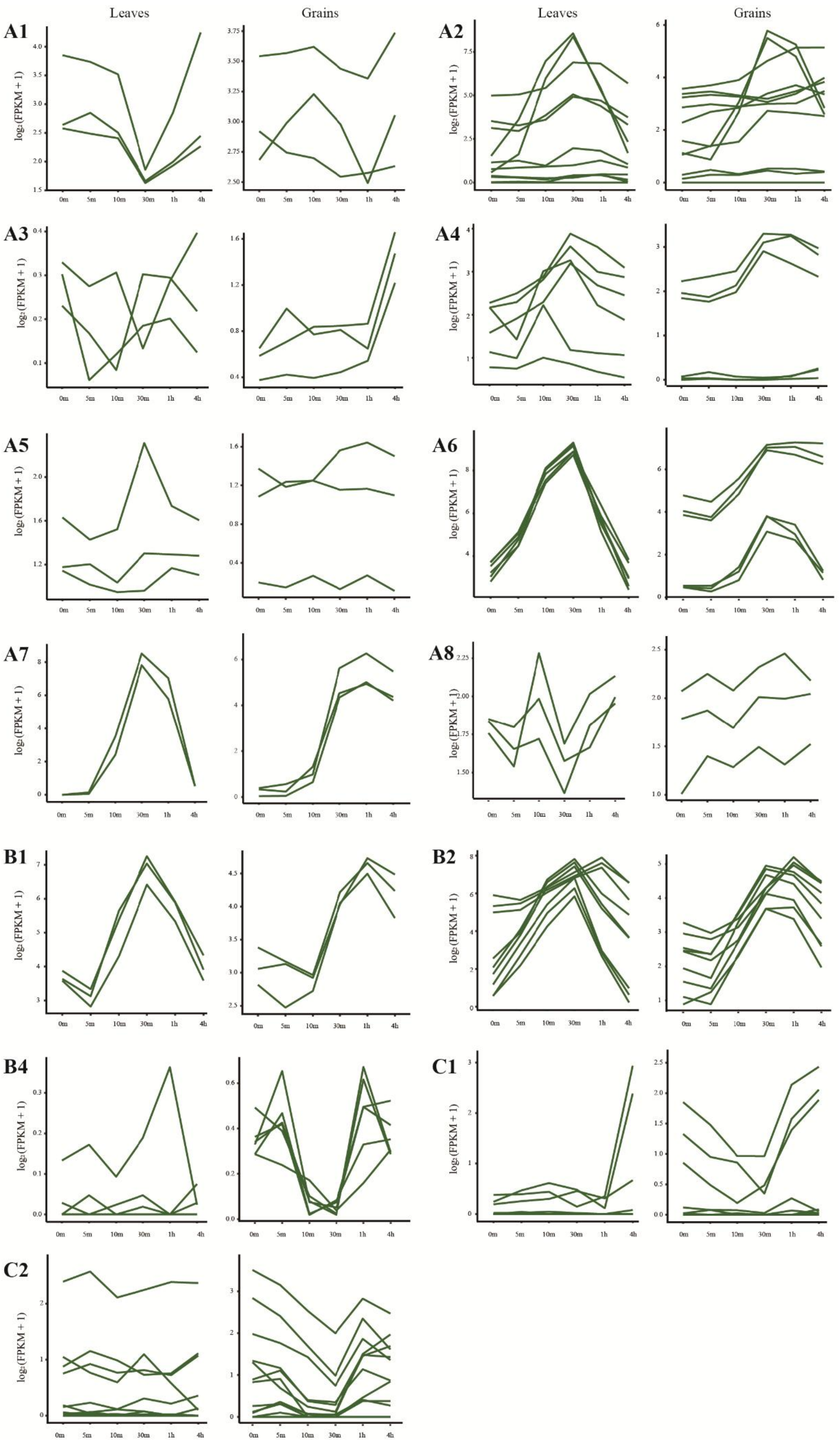
Leaves
Grains
Leaves
Grains
A1
A2
A3
A4
A5
A6
A7
A8
B1
B2
B4
C1
C2
log₂(FPKM + 1)
0m
5m
10m
30m
1h
4h

Supplemental Figure 11. The expression variation patterns of each member of each HSF subfamily. The *x*-axis represents treatment time points and the *y*-axis represents log2-transformed FPKM expression values. The symbols in the upper left indicate the subfamilies of HSFs.

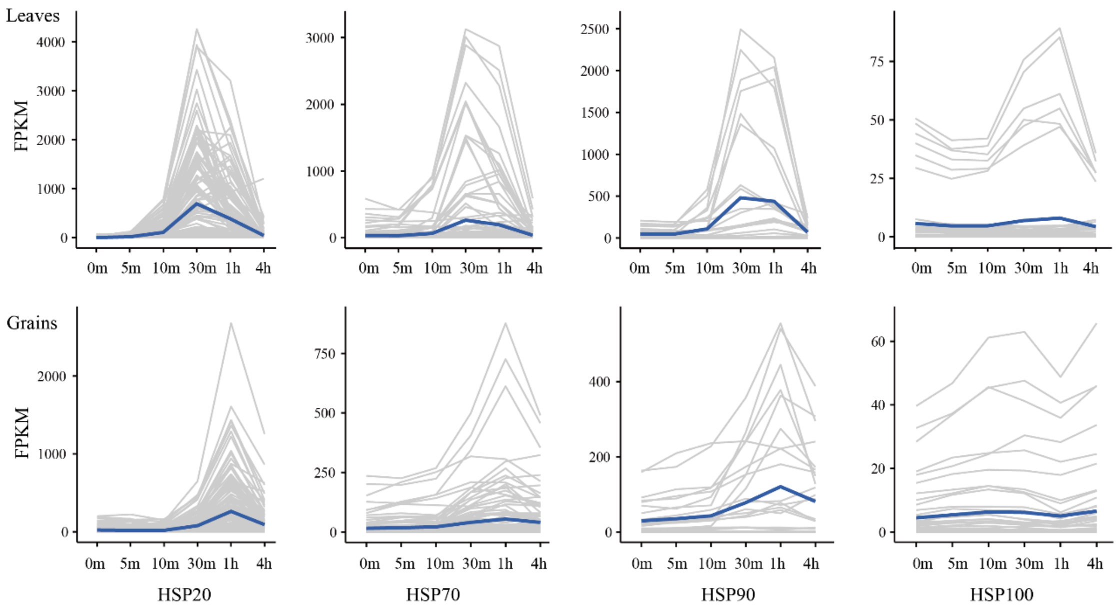

Supplemental Figure 12. The expression variation patterns of each member of the HSP20, HSP70, HSP90 and HSP100 families. The *x*-axis represents treatment time points and the *y*-axis represents expression values. The blue lines represent the median values at each time point.

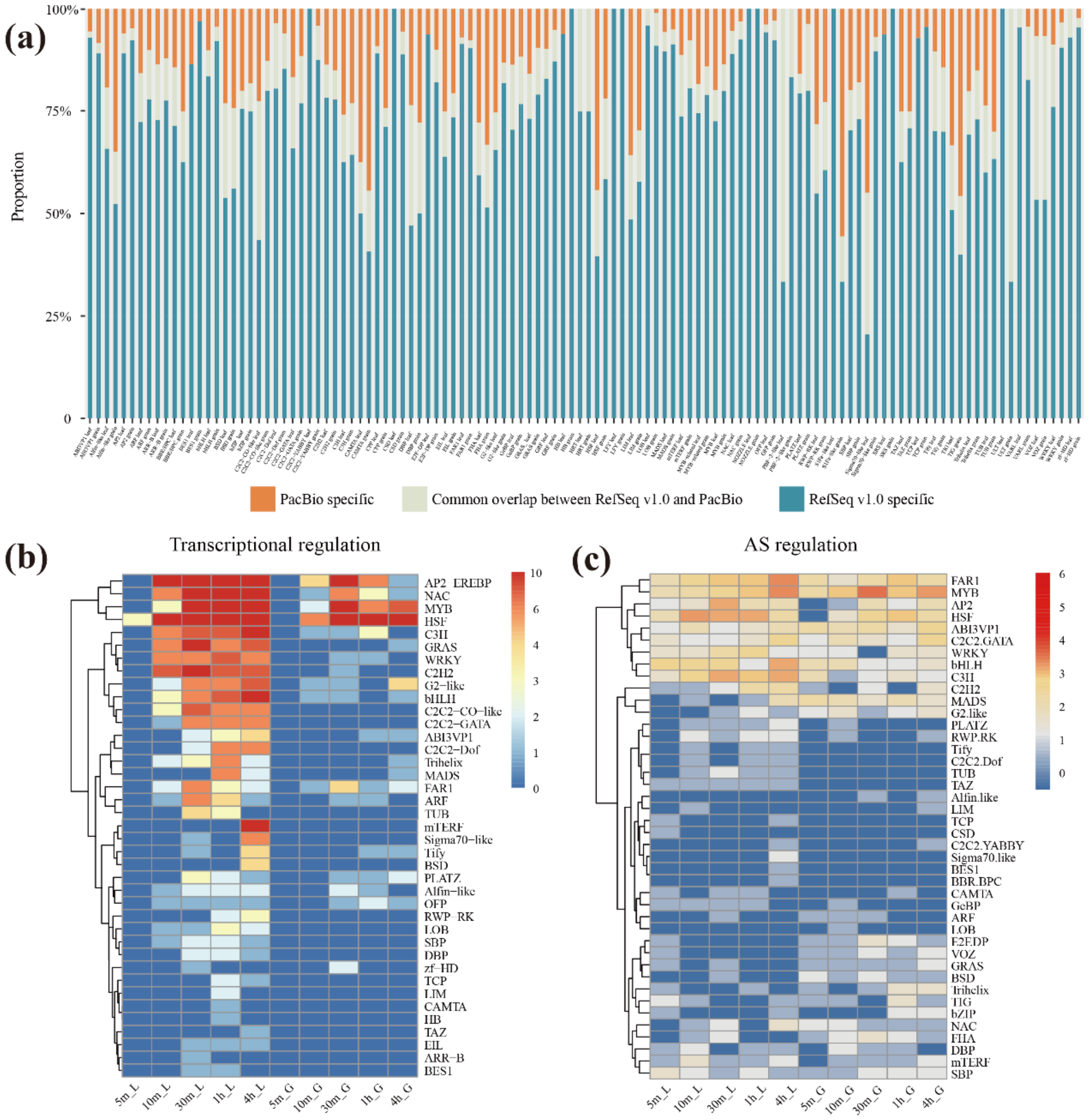

Supplemental Figure 13. HS-responsive TFs. (a) Comparison of novel TF isoforms identified from the PacBio data with the annotated isoforms of TFs in the IWGSC RefSeq v1.0. (b) Heatmap showing the number of DEGs in each TF family. The *x*-axis represents HS-treated samples and the *y*-axis represents the TF families. “L” and “G” in the sample names represent leaf and grain, respectively. (c) Heatmap showing the number of DSGs in each TF family. The TFs were identified based on the PlnTFDB 3.0 (http://plntfdb.bio.uni-potsdam.de/v3.0/).

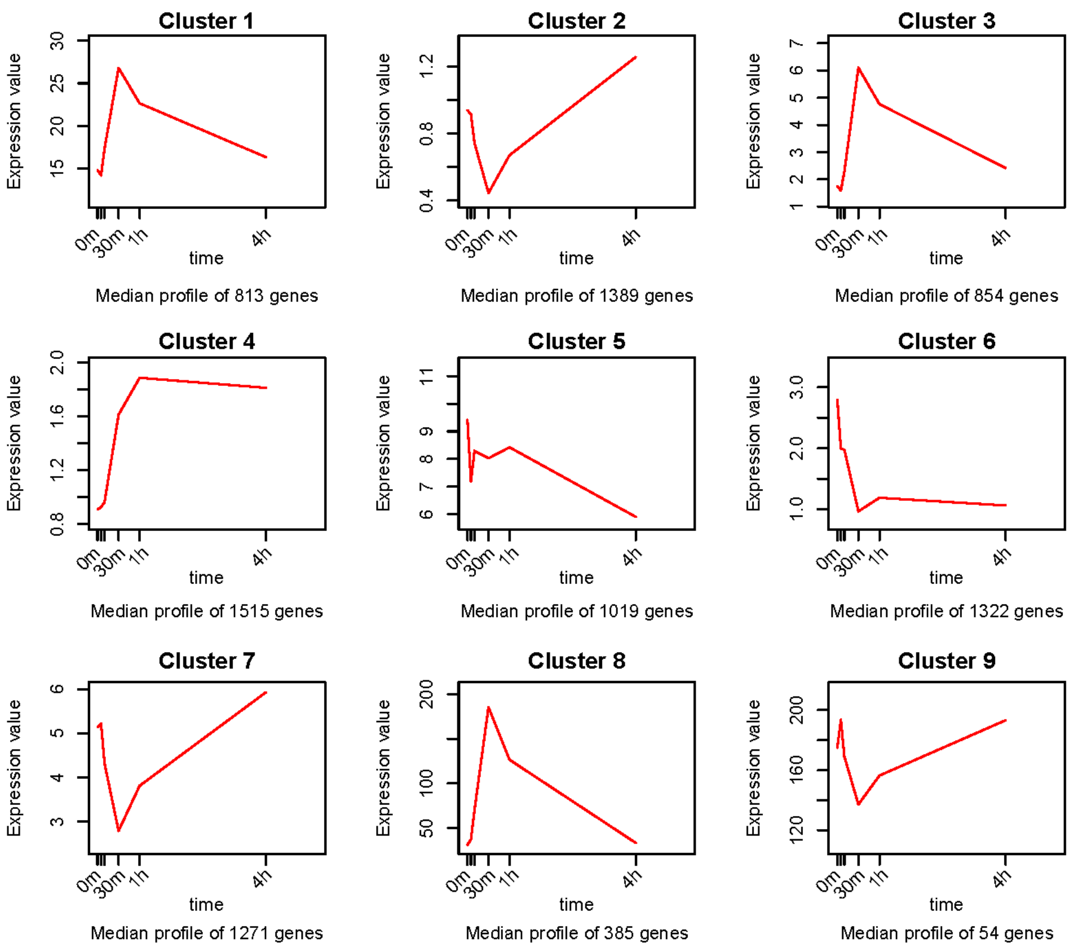

Supplemental Figure 14. The thermal conductivity and signaling duration in leaves. The DEGs in leaves were imported into the maSigPro (https://www.bioconductor.org/packages/release/bioc/html/maSigPro.html) to perform a time series analysis and classify the DEGs into different clusters, in each of which genes have similar expression profiles. The line present the expression profile of the genes in each cluster.

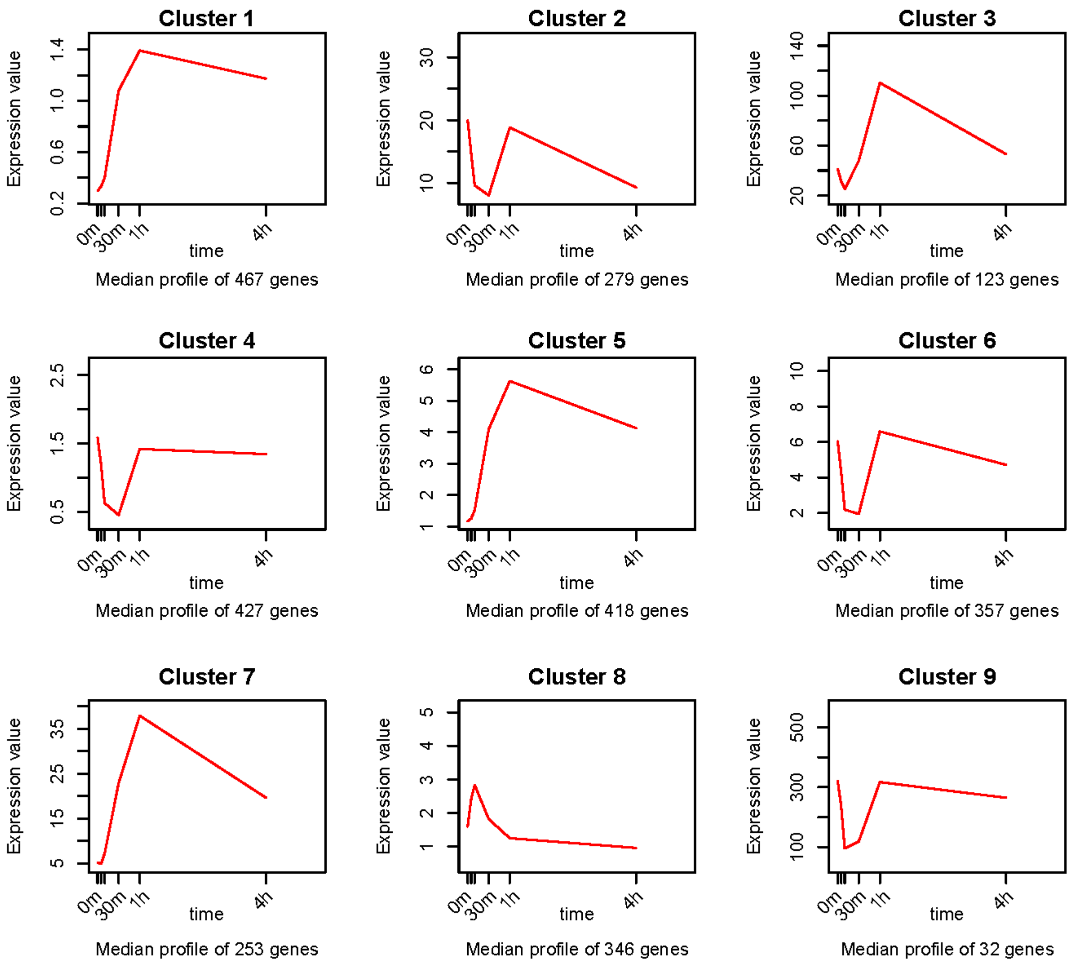

Supplemental Figure 15. The thermal conductivity and signaling duration in grain. The DEGs in leaves were imported into the maSigPro (https://www.bioconductor.org/packages/release/bioc/html/maSigPro.html) to perform a time series analysis and classify the DEGs into different clusters, in each of which genes have similar expression profiles. The line present the expression profile of the genes in each cluster.

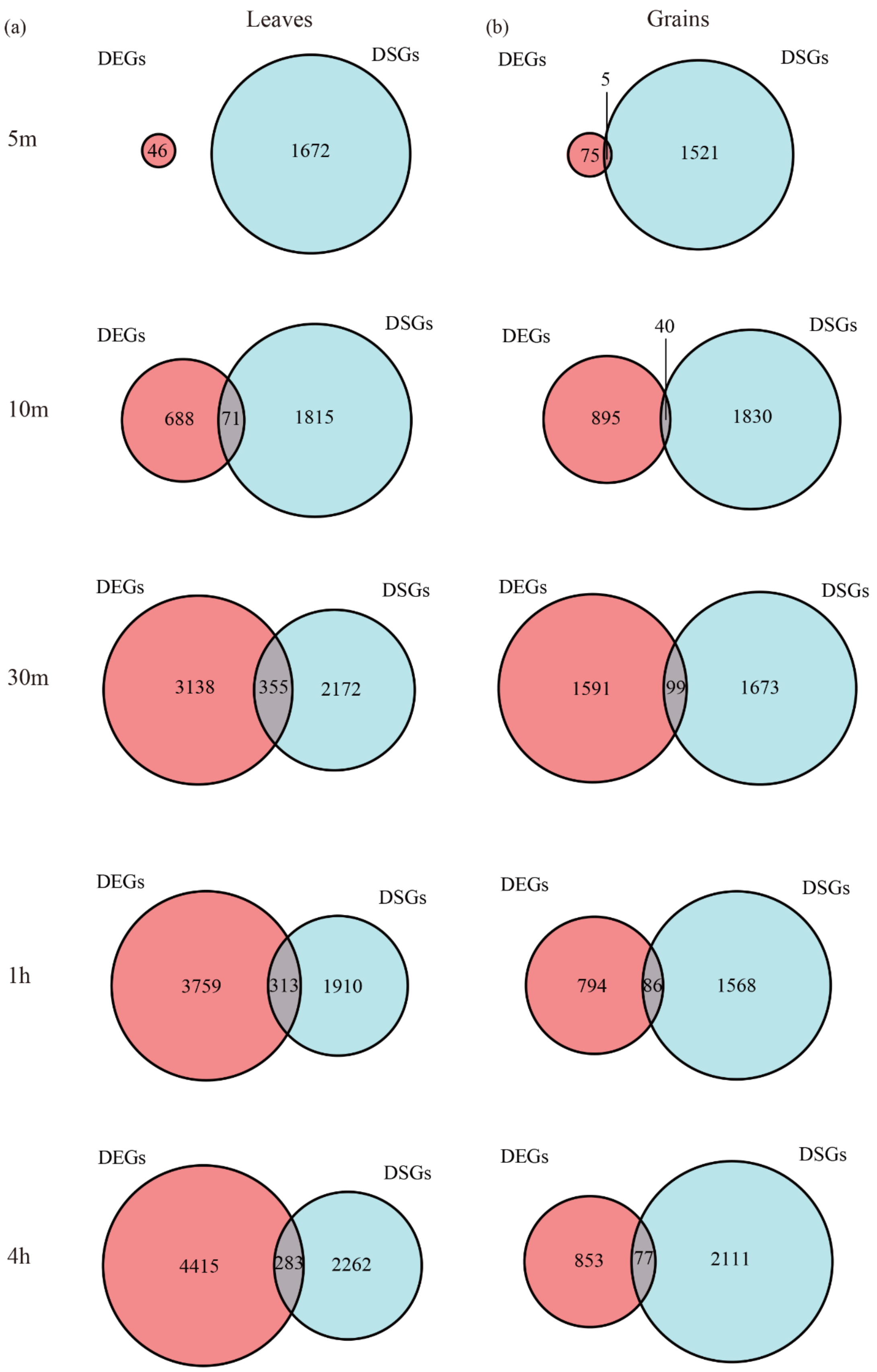

Supplemental Figure 16. Comparison of DEGs and DSGs at each HS treatment time point in leaves (a) and grains (b), respectively.

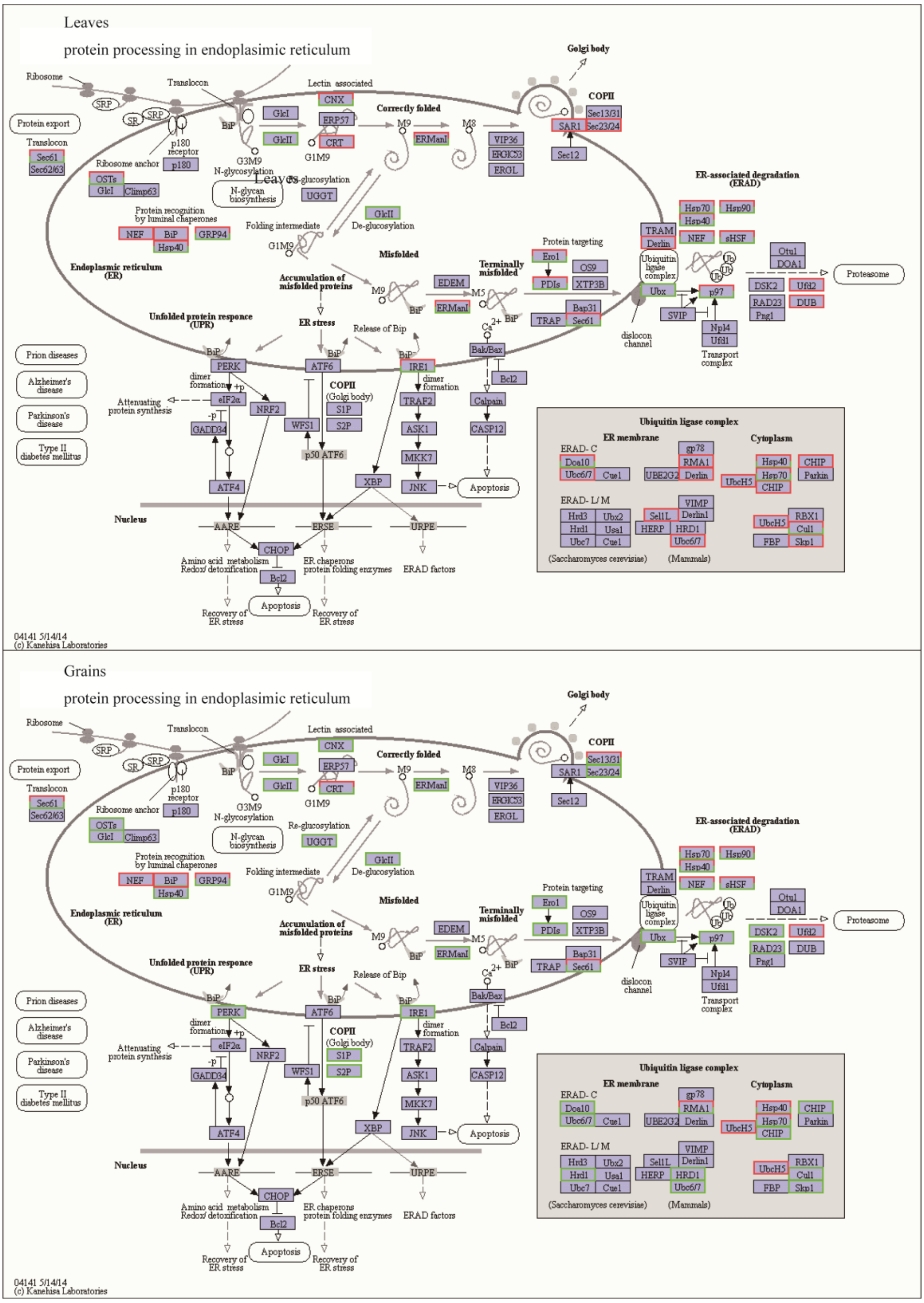

Supplemental Figure 17. Schematic representation of the protein processing in endoplasmic reticulum pathway exported from the KEGG database. The genes with red border and the green border represent the genes which response to HS with

transcriptional regulation and AS regulation, respectively. The genes with half red and half green border represents the genes which response to HS with both transcriptional regulation and AS regulation.

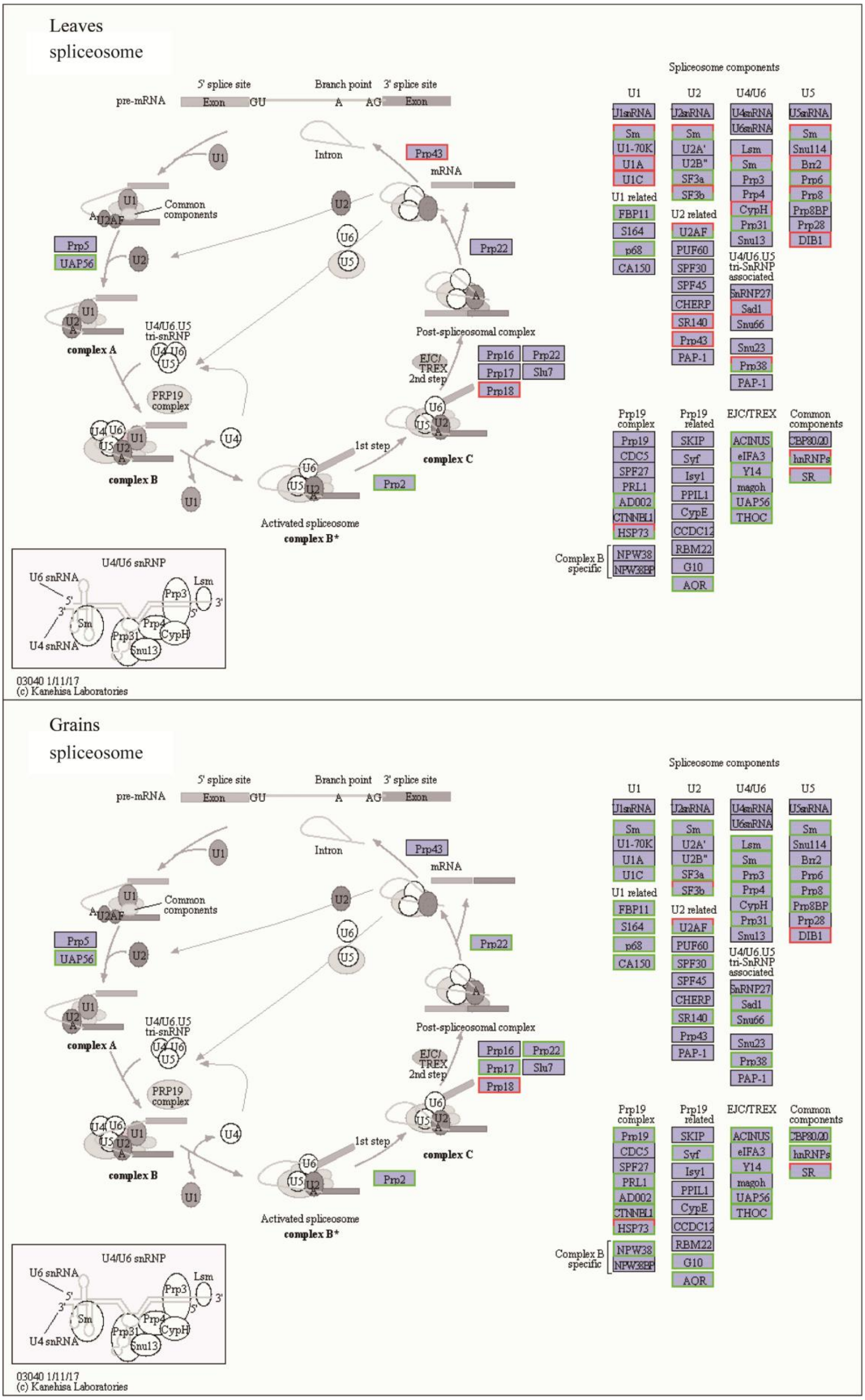

Supplemental Figure 18. Schematic representation of the spliceosome pathway

exported from the KEGG database. The genes with red border and the green border represent the genes which response to HS with transcriptional regulation and AS regulation, respectively. The genes with half red and half green border represents the genes which response to HS with both transcriptional regulation and AS regulation.

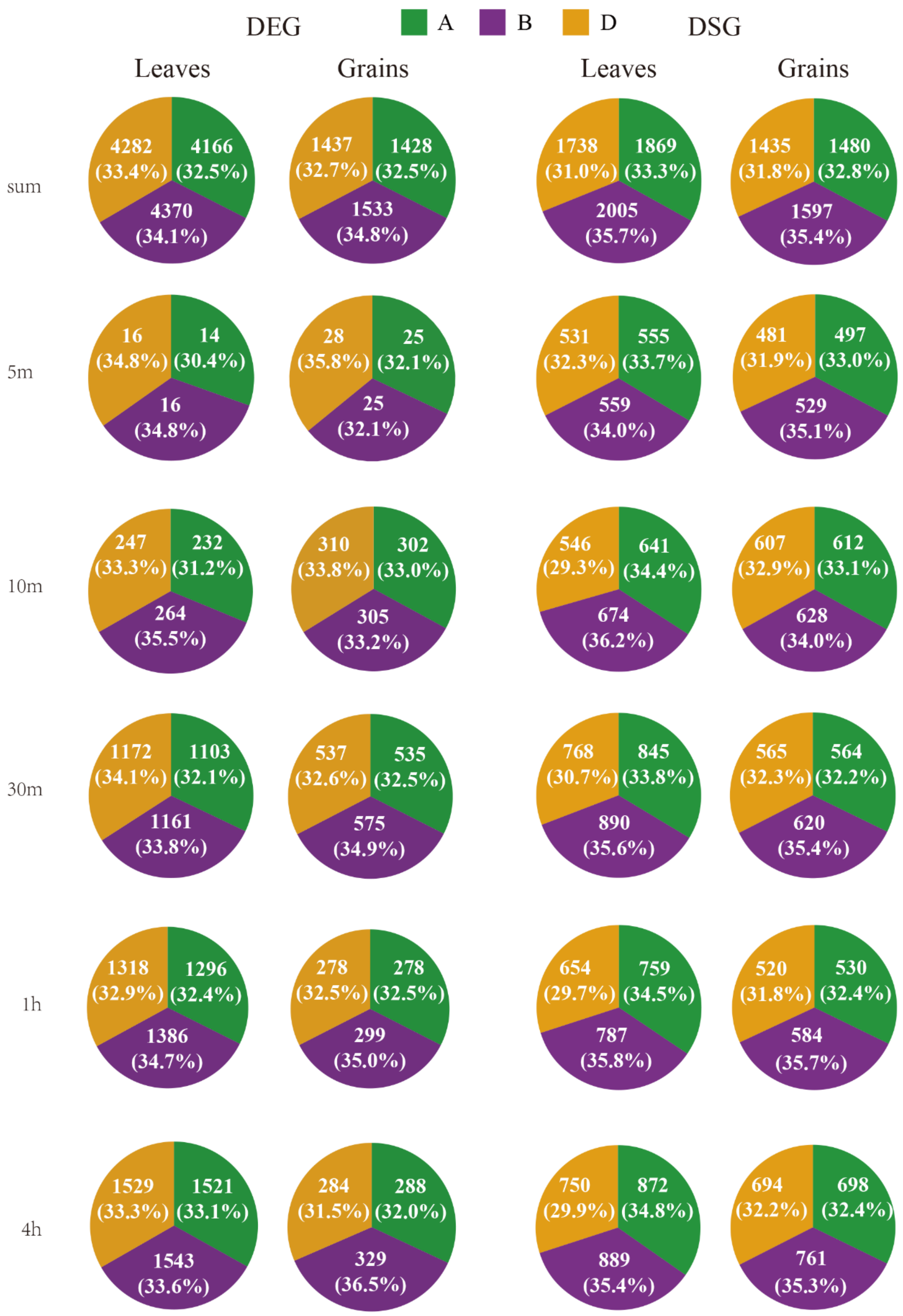

Supplemental Figure 19. Distribution of DEGs and DSGs among the three wheat subgenomes at each HS treatment time point. The left symbols represent HS treatment time points.

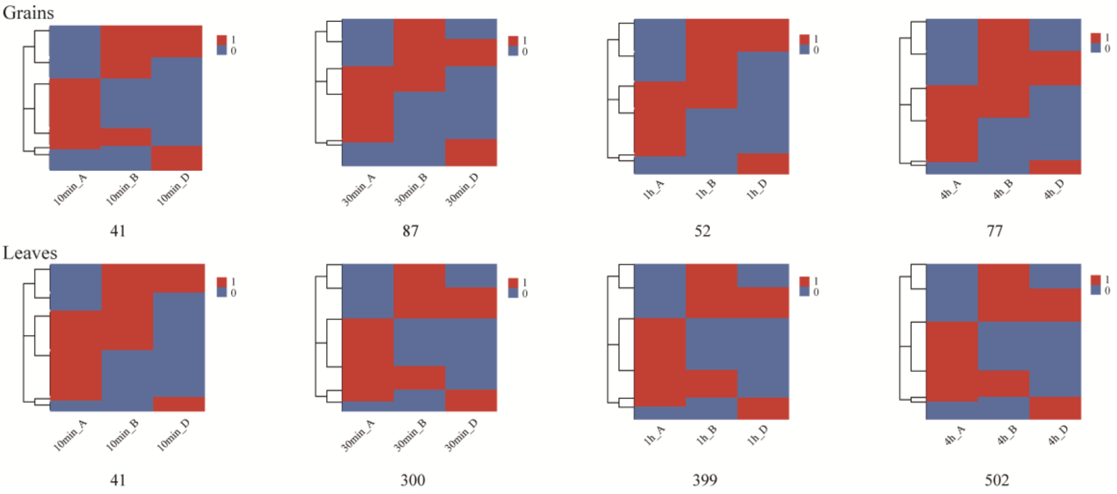

Supplemental Figure 20. Heatmaps display the DEGs in each triplet at each time point. Only the triplets that contain DEGs were displayed for each time point. By calculating the ratio of the fold change between the A-, B- and D-homeologues, the symbol "1" indicates that the gene exhibits differential HS responses according to the criteria of a 1.5-fold change. The number at the bottom of each heatmap represents the triplet number in each heatmap.

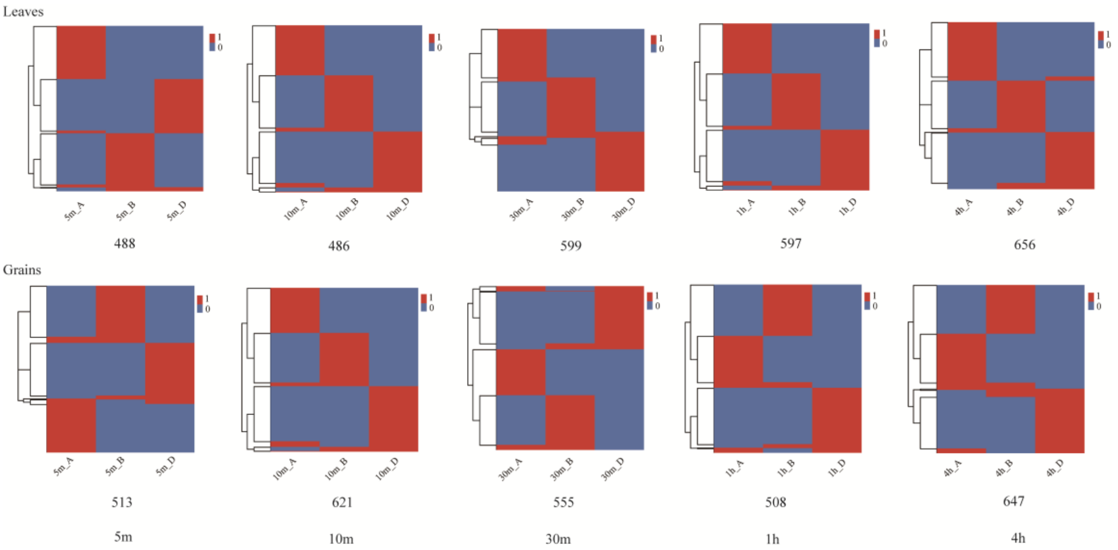

Supplemental Figure 21. Heatmaps display the DSGs in each homologous triplet at each time point. Only the triplets that contain DSGs were displayed for each time point. The symbol "1" indicates that the gene was a DSG and the symbol "0" indicates that the gene was not a DSG.

Supplemental Table 1. General properties of the reads produced by Illumina sequencing and PacBio sequencing.

Supplemental Table 2. The alignment of FLNC reads with the IWGSC RefSeq v1.0.

Supplemental Table 3. The gene and isoform annotations of the IWGSC RefSeq v1.0 and PacBio data.

Supplemental Table 4. The genes and isoforms identified in the PacBio data.

Supplemental Table 5. The BLAST hits of the newly discovered transcripts among the public databases.

Supplemental Table 6. The domain annotation and enrichment of newly identified loci in InterPro database.

Supplemental Table 7. The ratio of repeat sequences in newly discovered gene regions.

Supplemental Table 8. The annotation of the newly discovered loci.

Supplemental Table 9. The list of lncRNAs and their HS responses.

Supplemental Table 10. The list of DEGs.

Supplemental Table 11. The KEGG and GO enrichment analysis of genes which show different HS response between leaves and grains.

Supplemental Table 12. The list of DSGs.

Supplemental Table 13. The number and ratio of the four splicing modes in all PacBio isoforms or in the isoforms generated by DSGs.

Supplemental Table 14. The classification of HSFs and their response to HS.

Supplemental Table 15. The classification of HSPs and their response to HS.

Supplemental Table 16. The KEGG and GO enrichment analysis of DEGs.

Supplemental Table 17. The KEGG and GO enrichment analysis of DEG clusters in leaves and grain.

Supplemental Table 18. The KEGG and GO enrichment analysis of DSGs.

Supplemental Table 19. The KEGG and GO enrichment analysis of DEG- and DSG-specific genes and overlapping genes between DEGs and DSGs.

Supplemental Table 20. The list of identified homologous triplets.

Supplemental Table 21. The KEGG and GO enrichment analysis of distinct categories based on the differential HS responses between the A-, B- and D-homeologues.
